# Cervicovaginal microbiome and natural history of *Chlamydia trachomatis* in adolescents and young women

**DOI:** 10.1016/j.cell.2024.12.011

**Published:** 2025-01-15

**Authors:** Mykhaylo Usyk, Luke Carlson, Nicolas F. Schlecht, Christopher C. Sollecito, Evan Grassi, Fanua Wiek, Shankar Viswanathan, Howard D. Strickler, Anne Nucci-Sack, Angela Diaz, Robert D. Burk

**Affiliations:** 1 Departments of Microbiology and Immunology, Albert Einstein College of Medicine, Bronx, New York, NY, USA; 2 Department of Pediatrics (Genetic Medicine), Albert Einstein College of Medicine, Bronx, New York, NY, USA; 3 Department of Pediatrics, Mount Sinai Adolescent Health Center, Icahn School of Medicine at Mount Sinai, Manhattan, New York, NY, USA; 4 Department of Epidemiology and Population Health, Albert Einstein College of Medicine, Bronx, New York, NY, USA; 5 Department of Cancer Prevention & Control, Roswell Park Comprehensive Cancer Center, Buffalo, NY, USA; 6 Department Obstetrics and Gynecology and Women’s Health, Albert Einstein College of Medicine, Bronx, New York, NY, USA; 7 Deceased; 8 Lead contact

## Abstract

This study investigated the cervicovaginal microbiome’s (CVM’s) impact on *Chlamydia trachomatis* (CT) infection among Black and Hispanic adolescent and young adult women. A total of 187 women with incident CT were matched to 373 controls, and the CVM was characterized before, during, and after CT infection. The findings highlight that a specific subtype of bacterial vaginosis (BV), identified from 16S rRNA gene reads using the *molBV* algorithm and community state type (CST) clustering, is a significant risk factor for CT acquisition. A microbial risk score (MRS) further identified a network of bacterial genera associated with increased CT risk. Post treatment, the CVM associated with CT acquisition re-emerged in a different subset of cases leading to reinfection. Additionally, the analysis showed a connection between post-treatment CVM and the development of pelvic inflammatory disease (PID) and miscarriage, further underscoring the CVM’s contributing role to incident CT natural history and highlighting its consideration as a therapeutic target.

## In brief

By analyzing the cervicovaginal microbiome across the entire natural history of incident *Chlamydia trachomatis* (CT) infection among Black and Hispanic adolescent and young adult women in NYC, researchers can better understand associations between the cervicovaginal microbiome and CT infection risk, likelihood of reinfection, pelvic inflammatory disease, and miscarriage.

## Graphical Abstract

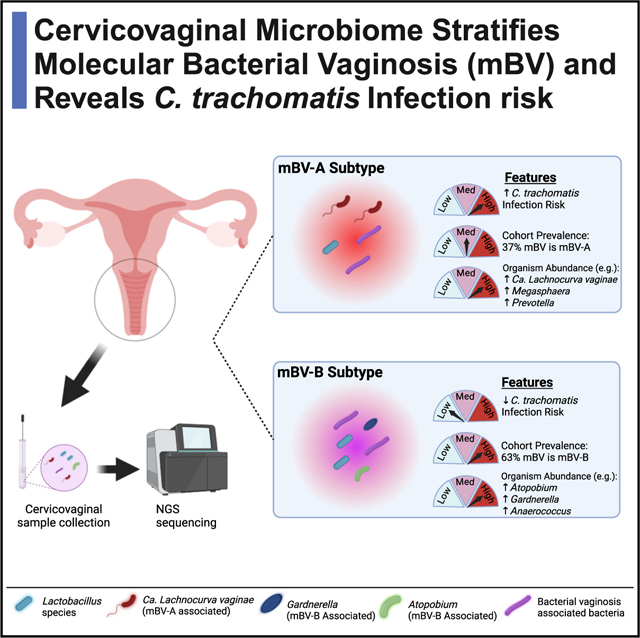

## INTRODUCTION

*Chlamydia trachomatis* (CT) is the most common bacterial sexually transmitted infection (STI), accounting for over 130 million cases worldwide in 2019, and has continued to rise in incidence over the past 40 years.^1^ The large number of cases significantly impacts public health, with approximately 20% of infected women experiencing sequelae—including pelvic inflammatory disease (PID), chronic pelvic pain, ectopic pregnancy, and infertility—and their newborn infants experiencing conjunctivitis and pneumonia.^2,3^ Moreover, over half of all incident STIs, including CT, affect adolescents and young adults (AYAs), and there are considerable racial and ethnic disparities in rates of CT infections—Black and Hispanic AYAs have 5-fold higher rates compared with non-Hispanic White AYAs.^4,5^ There is also a socioeconomic and racial disparity in population research on CT.^6^ Therefore, it is important to identify risk factors for CT to facilitate interventions to reduce morbidity and the substantial public health burden of infections.

Currently, there are several well-established risk factors for CT acquisition, including multiple sex partners, prior STIs and/or CT history, younger age (<25 years), and poor education.^7–9^ In addition, bacterial vaginosis (BV), defined as vaginal dysbiosis that is often accompanied by inflammation and vaginal discharge,^10–12^ has also been proposed as a risk factor for CT acquisition.^13,14^ However, it is difficult to disentangle the correlation between CT acquisition and BV since both are influenced by sexual behaviors.^15–17^ This is further complicated by the fact that both CT infections and BV are commonly asymptomatic, making true estimation and tracking of this association difficult.^7^

The human vaginal tract contains multiple species of bacteria and fungi that collectively comprise the cervicovaginal microbiome (CVM), which is critical in maintaining genital tract health.^18^ CVMs either predominantly contain species of *Lactobacillus* (e.g., *L. crispatus*, *L. iners*, *L. gasseri*, or *L. jensenii*) or exist in a polymicrobial state.^19^ The roles of these lactobacilli include protection against CT via such mechanisms as secretion of lactic acid.^20^ Additionally, species of *Lactobacillus* have been proposed to inhibit CT propagation by eliminating vaginal tryptophan, with *L. crispatus* having multiple anti-CT properties.^3^ BV is also considered a risk factor for CT, but the overlap between risk factors for BV and CT makes it difficult to determine whether it is the shared behavioral/demographic factors that are responsible for the association between BV and CT.^13^ However, since no single microbial agent of BV has been identified, there is equipoise on whether BV can be considered an STI.^21^

There are a number of studies that have investigated the characteristics of the CVM and/or BV prior to the development of a CT infection.^14,22,23^ Nevertheless, the temporal relationship and molecular characteristics of the CVM in relation to CT infections are not fully understood, particularly given the dynamic nature of the CVM.^24^ Moreover, it is important to study CT and BV in a context that controls for similar risk factors (e.g., sexual behavior) in order to eliminate confounding due to shared causal factors.^25^

In this report, we present data from a large cohort of sexually active Black and Hispanic AYA women (*n* = 560) evaluating the CVM before, during, and after an incident CT infection and subsequent sequelae. We employed a nested case-control design using risk-set sampling matched on age and CT history within a large clinical community open dynamic cohort study.^26^ To investigate the temporal associations between the CVM and CT, we tested cervicovaginal samples collected approximately 6 months prior to (t_−1_), at the time of (t_0_), and 6 months after treatment (t_+1_) of the incident detection of CT infection (case women). In addition, we tested post-treatment follow-up samples to assess the impact of antibiotic treatment of CT infection on the CVM (t_+1_) and sequelae. We present the molecular characterization of the CVM using a previously published *molBV* pipeline^27^ that converts 16SV4 rRNA gene sequencing into a molecular Nugent-like score and also determined categorical community state types (CSTs). Integration of molecular BV (mBV) and CSTs identified two forms of BV, one of which was highly predictive of acquiring CT and sequelae. This information could have clinical implications.

## RESULTS

### Participant characteristics

**Figure 1.**
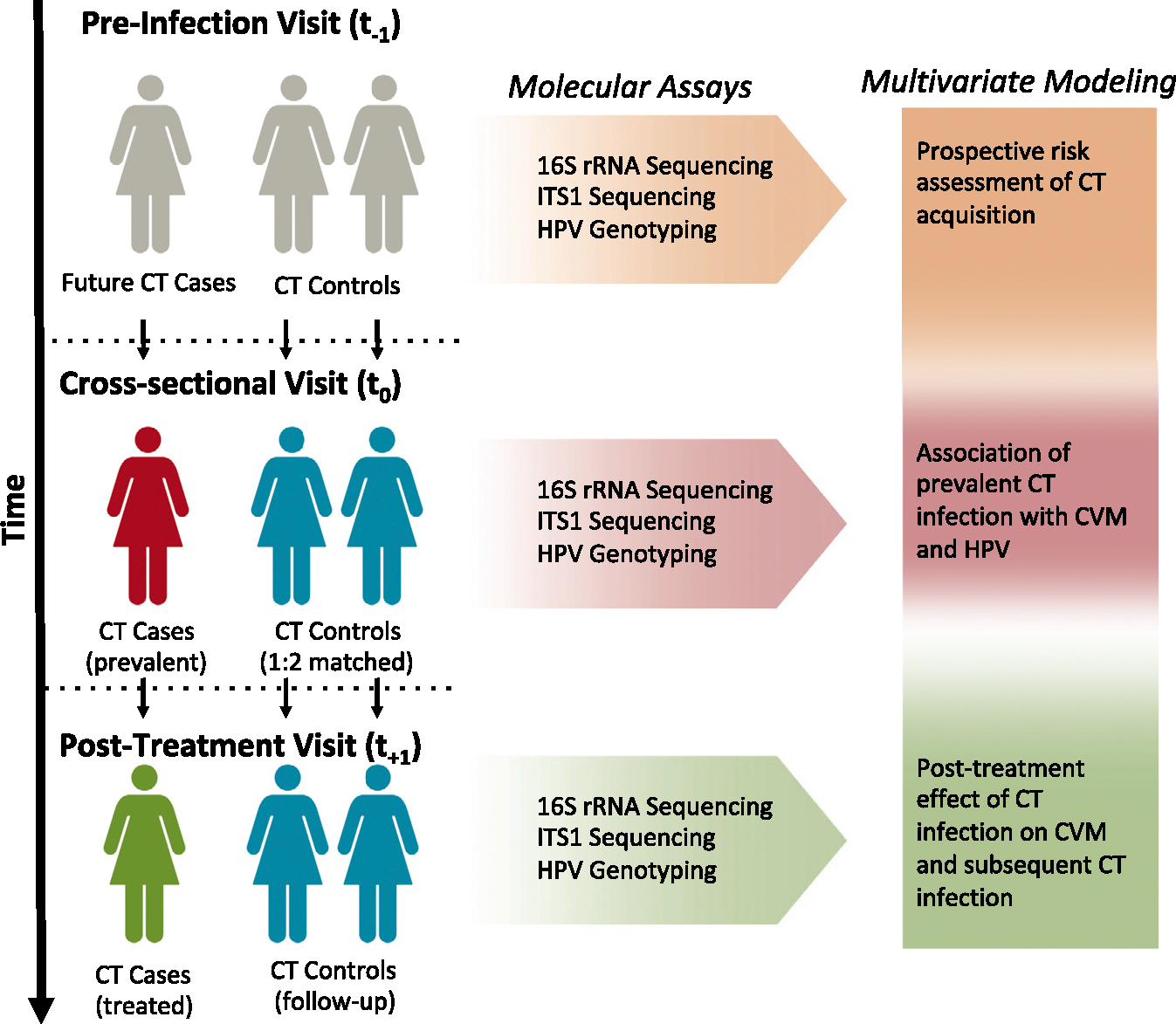
Prospective CT natural history study design overview Case subjects (*n* = 187) were selected based on identification of an incident CT infection diagnosed during routine clinical screening with the Gen-Probe APTIMA test. Controls (*n* = 373) were matched to cases (2:1, respectively) at the time of incident CT infection based on age, date of study enrollment, and prior history of CT infection. Retrospective cervicovaginal swab samples were retrieved from prior visits for all participants (approximately 6 months for both cases and controls). Post-treatment samples were collected in 504/560 participants (approximately 8 months after case CT infection treatment). All cervical samples underwent 16SV4 rRNA and ITS1 amplicon sequencing for assessment of bacteria and fungi/eukaryotes, respectively. HPV genotyping was analyzed using data from the parent study and included high-risk (oncogenic) HPV types—HPV16, −18, −31, −33, −35, −39, −45, −51, −52, −56, −58, and −59. Multivariate modeling was used to determine (1) prospective risk of CT acquisition (at t_−1_), (2) the association of CT infection and the CVM (at t_0_), and (3) residual effects of treated CT infection on the CVM (at t_+1_).

The study design and cohort characteristics are shown in Figure 1 and Table 1, respectively. The study sample was selected by matching incident CT cases (*n* = 187) to controls (*n* = 373) using 1:2 matching on age, enrollment year, study follow-up time, and prior history of CT infection. Overall, the cohort participants were all sexually active, were an average of 20 years old (range 13–21 years), were attending school, and had similar sexual risk behavior scores (SRBSs)^28^ (see Table S1 for a comparison of the individual behavioral variables across cases and controls).

**Figure 2.**
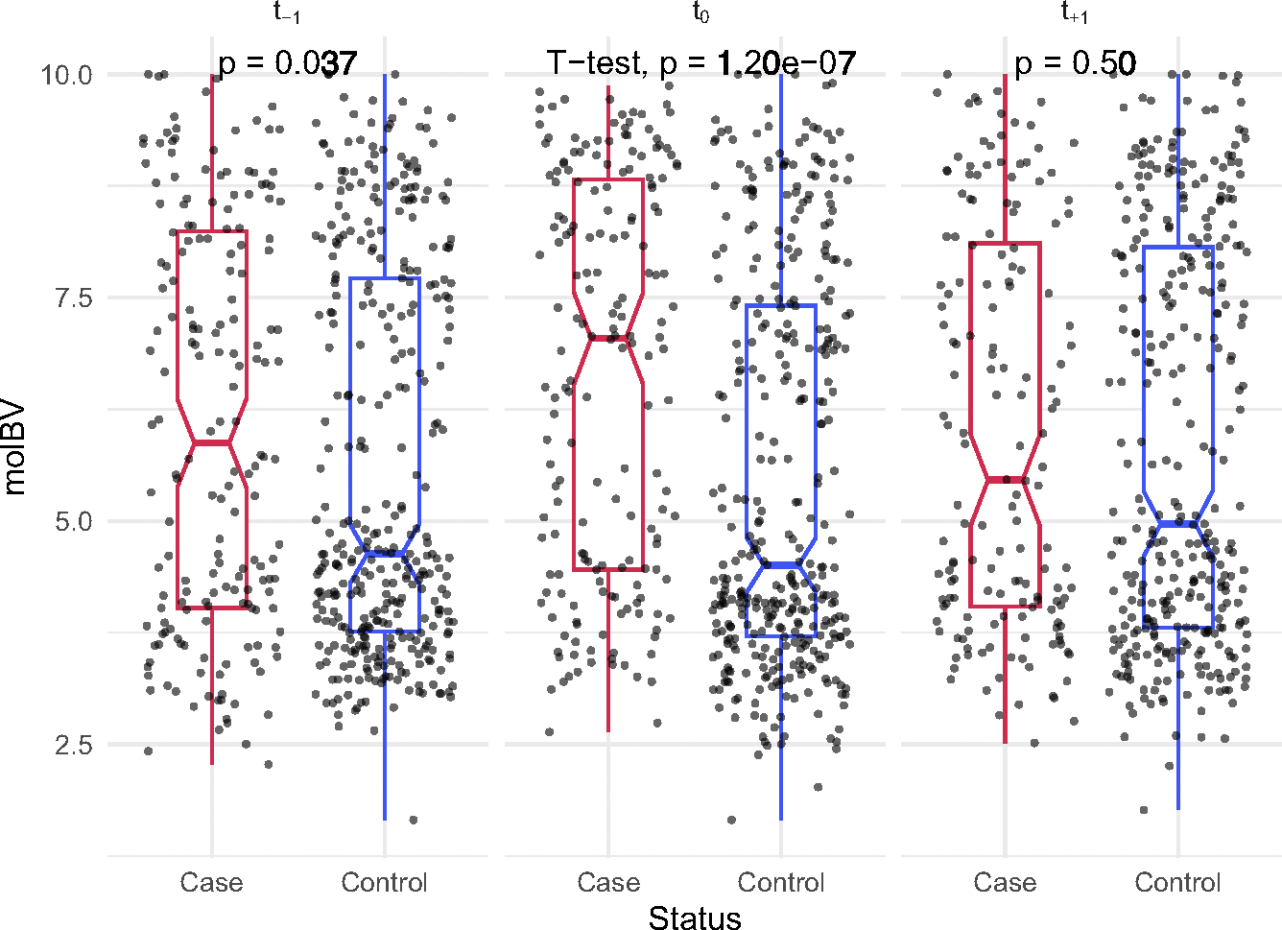
Distribution of *molBV* scores across pre-, incident-, and post-CT visits Figure shows the molecular Nugent score (termed *molBV*) distributions across the three visits (i.e., pre-infection, t_−1_, cross-sectional, t_0_, and post-treatment, t_+1_) with *p* values shown above box-plots based on Wilcoxon rank sum test applied to the case-control status established at t_0_. *molBV* is a quantitative measure of BV (ranging from 0–10, with higher values representing a more BV-like state). In *molBV* analyses, the prospective t_−1_ and cross-sectional t_0_ visits showed significant association with incident CT infection in cases. See also Figures S1 and S5.

We analyzed the *molBV* scores (i.e., a Nugent-like score of 1–10 using a *molBV* algorithm^27^) given the well-known association of BV and CT^25^ (Table 1). We used a molecular assessment of the CVM since it provides an objective characterization of BV that is independent of whether a participant has BV-like symptoms.^19^ Compared with the controls, the estimated average (median) *molBV* scores were higher among the cases at the cross-sectional t_0_ visit (Figure 2, *p* < 0.001). Interestingly, there was also a significant increase in the *molBV* scores among future cases at the pre-infection t_−1_ visit (*p* = 0.037) but not at the post-treatment follow-up visit t_+1_ (*p* = 0.50), suggesting that CVM dysbiosis may be a risk factor for CT acquisition. Features of CSTs also showed a difference between cases and controls at the incident CT t_0_ visit and the pre-infection t_−1_ visit.

### Risk factors for development of incident CT infection (pre-infection t_−1_ visit)

To determine features of the CVM that were risk factors for incident CT infection, we analyzed the different facets of the microbiome (Figures 2 and S1). In terms of α diversity, bacterial evenness and richness were significantly elevated in AYA women before acquiring CT compared with controls (see Figure S1A, visit t_−1_ sample Shannon, *p* = 0.04). Similarly, the overall community composition of bacteria as measured by β diversity also revealed a significant difference prior to the acquisition of CT (see Figure S1B, R^2^ = 0.011, *p* = 0.025). However, neither diversity measure for the fungal communities was associated with incident CT (see Figures S1C and S1D).

Identifying BV-like features, e.g., increased microbial diversity^27,29^ and elevated *molBV* scores at visit t_−1_ (Figure 2), supported the association between BV and prospective risk of CT (detected at visit t_0_). In order to further evaluate BV and risk of incident CT, we used multivariable conditional logistic regression and categorical states of mBV as shown in the pre-infection visit model (t_−1_) in Table 2. mBV (mBV-positive, *molBV* score 7–10) was significantly associated with development of CT (odds ratio [OR] = 1.62, 95% confidence interval [CI]: 1.01–2.59, *p* = 0.04), whereas mBV-intermediate (*molBV* score >3–<7) also had an increased risk for CT but did not achieve statistical significance (OR = 1.46, 95% CI: 0.90–2.38, *p* = 0.12). There was no evidence that high-risk human papillomavirus (HR-HPV) infection at t_−1_ was an independent risk factor for incident CT (*p* = 0.40). To examine the extent to which the participants’ sexual behavior was acting through BV leading to subsequent incident CT infection, we performed a causal mediation analysis (see “mediation analysis” in the STAR Methods). Results of the mediation analysis revealed that differences in the SRBSs were not associated with incident CT, nor was the potential association with SRBSs mediated by BV. This result is likely due to the homogeneity of sexual behaviors in the study cohort.

### Categorizing the CVM using CSTs and *molBV* (pre-infection t_−1_ visit)

A complementary approach to characterize the CVM employs 16S rRNA amplicon sequencing is to define bacterial cervicovaginal microbiome CSTs, as previously described.^30^ Table 1 shows the counts and proportions of CSTs across study visits. To incorporate CSTs into a Nugent-like clinical framework of BV, we first compared the distribution of *molBV* scores with CSTs and found that the *Lactobacillus*-dominated CSTs showed consistently low *molBV* scores, whereas mBV-positives (i.e., *molBV* 7–10) were primarily limited to CST-IV-A and CST-IV-B (Figure S2). CST-IV-A is characterized by the species *Candidatus Lachnocurva vaginae* (previously known as BVAB1), whereas CST-IV-B is associated with *Atopobium vaginae*.^30^ Modeling the CSTs as risk factors for incident CT revealed that surprisingly, only CST-IV-A showed a prospective association with incident CT (OR = 2.46, 95% CI: 1.16–5.19, *p* = 0.020), and CST-IV-B showed elevated risk but did not reach statistical significance (OR = 1.44, 95% CI: 0.77–2.70, *p* = 0.25) (Table 2). To incorporate CST definitions into our categorical mBV analysis, we categorized mBV-positive participants at the t_−1_ visit into mBV-A subtype if they concurrently had CST-IV-A and into mBV-B subtype if they did not have CST-IV-A. Table 3 (see model A, pre-infection visit) shows the full model for the mBV subtypes. The analysis revealed that only participants with mBV-A showed a statistically significant elevated risk for acquiring a CT infection (OR = 2.38, 95% CI: 1.21–4.69, *p* = 0.012). Given that mBV-A showed increased risk for CT, we further explored the features of this BV subtype compared with mBV-B using analysis of composition of microbes (ANCOM). Results confirmed that *Candidatus Lachnocurva vaginae* was the top species associated with mBV-A, and levels of this species showed an area under the curve (AUC) for mBV-A classification of 0.93 (Figure S3). Direct analysis of the levels of *Candidatus Lachnocurva vaginae* showed a 33-fold increase of *Candidatus Lachnocurva vaginae* in mBV-A when compared with mBV-B.

### Prospective risk of CT acquisition using a polymicrobial risk score (pre-infection t_−1_ visit)

**Figure 3.**
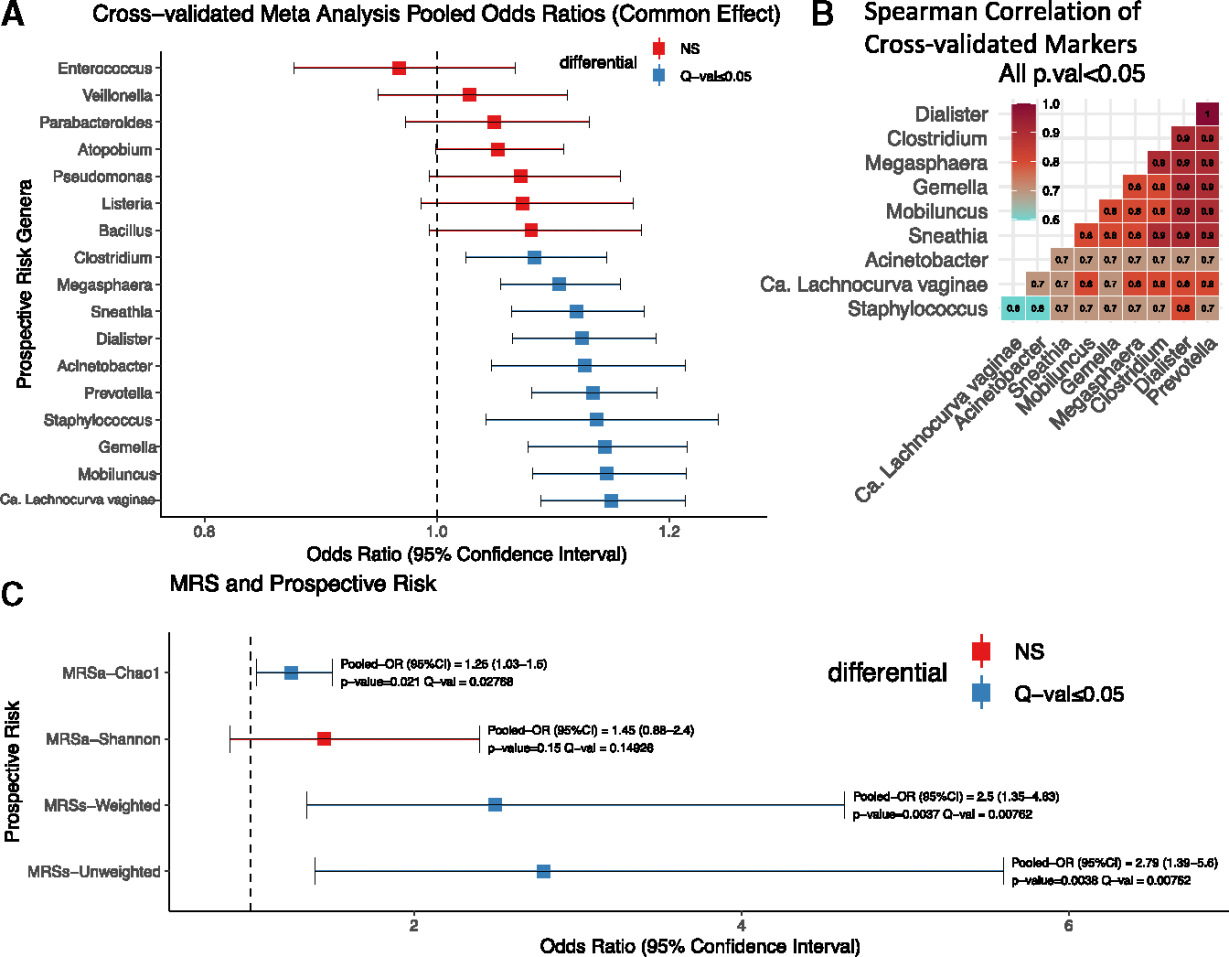
Cross-validation and bacterial community modeling of prospective bacterial risk factors for incident CT (A) The cross-validated combined common effects of all bacterial genera identified as significantly associated by ANCOM with prospective CT acquisition. Out of 18 genera identified, 10 were significant in the validation phase indicated in blue. The OR values represent the odds of incident CT infection per unit increase of each taxon (modeled as log ratios compared with *Lactobacillus*). (B) The correlation matrix for all genera confirmed to be associated with prospective CT acquisition with Spearman correlation values displayed within each cell. (C) Microbial risk scores (MRSs), including corresponding effect sizes, *p* values, and adjusted q values. A blue color denotes a q value ≤ 0.05, while red signifies a q value > 0.05. The OR values represent the odds of incident CT infection per unit increase of each continuous MRS score. See also Figures S3 and S4.

We evaluated whether individual taxa acted as independent risk factors or were part of a polymicrobial community for incident CT. First, we performed differential abundance analysis with ANCOM and 10-fold cross-validation (see Figure S4). Figure 3A shows the composite results for all bacteria found to be significantly associated with prospective CT acquisition by ANCOM. Following cross-validation, 10 taxa remained significant markers for CT acquisition, including *Candidatus Lachnocurva vaginae*, *Prevotella*, *Megasphaera*, and *Clostridium*, among other BV-associated bacteria.^31^ Matrix analyses suggested that these taxa belonged to a microbial network as they were highly correlated (pairwise correlations > 0.6, *p* < 0.001, Figure 3B). To further investigate how these taxa were collectively related to CT risk, we employed the microbial risk score (MRS) (which is analogous to a polygenic risk score^32^). Figure 3C presents the weighted analysis for CT acquisition per unit increase in MRS, OR = 2.50 (95% CI: 1.35–5.60, *p* = 0.0037). The values of the MRS risk estimates were considerably higher than individual bacterial risk estimates, indicating the importance of bacterial communities (see Figure 3).

### Perturbation of the CVM by CT: Cross-sectional associations of CT at the incident visit (t_0_ visit)

**Figure 4.**
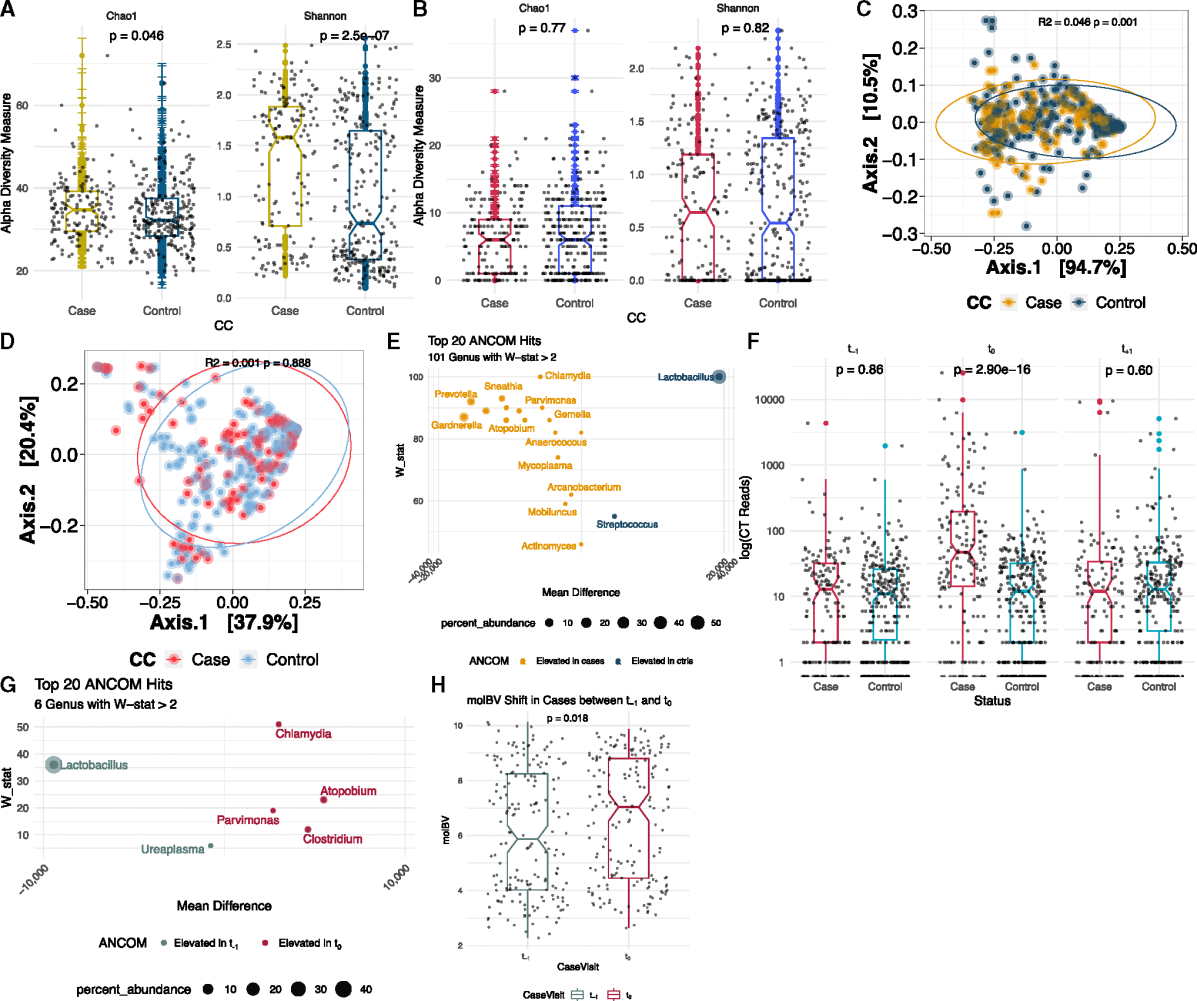
Association of CT infection and cervicovaginal microbiome (A and B) The bacterial and fungal α diversity at t_0_, respectively, with *p* values at the top of each panel calculated using Wilcoxon rank sum test. Note the y axis scales are different. (C and D) Principal coordinate analysis (PCoA) for bacteria and fungi at t_0_, respectively, with the R^2^ and *p* values calculated using a PERMANOVA test. (E) Results of ANCOM analysis that identifies differentially abundant genera associated with CT case status at t_0_. A total of 37 genera were identified to have a W-stat > 2 (FDR < 0.05 for all) with the top 20 genera presented in (E) (ranked based on W-stat). (F) Total recovery of *C. trachomatis* 16SV4 rRNA reads at each of the three study visits with Wilcoxon rank sum *p* values. (G) The genera altered as a result of CT acquisition in cases (i.e., t_−1_ vs. t_0_). (H) Distribution of *molBV* between CT cases at t_−1_ (red) and t_0_ (green).

The CVM bacterial α diversity (Figure 4A) was higher in the CT cases, whether based on the number of taxa (Chao1, *p* = 0.046) or taking evenness into account (Shannon, *p* = 2.50 × 10^−7^). Fungal α diversity was not associated with CT case status (Figure 4B). Similarly, the bacterial analysis showed differences with respect to composition measured by β diversity and CT case status (Figure 4C, R^2^ = 0.046, *p* = 0.001), whereas the fungal analysis did not (Figure 4D, R^2^ = 0.001, *p* value = 0.89).

Exploring differential abundance at the species level revealed a total of 37 bacterial species that were consistently different between CT cases and controls (Figure 4E). As anticipated, CT was the most differential bacteria, being higher in the cases than controls (W-stat = 41, false discovery rate [FDR] < 0.05), although only accounting for 0.054% of the total microbial biomass at the cross-sectional visit (t_0_). Considering all visits, cases had a spike in CT reads only at the t_0_ visit (*p* = 2.90 × 10^−16^) and not at the pre-infection t_−1_ (*p* = 0.60) or post-treatment t_+1_ visits (*p* = 0.86, Figure 4F). Based on the 16S rRNA V4 amplicon sequencing, we identified CT reads in 100/187 (53.5%) of cases compared with 61/373 (16.4%) of the controls at the incident infection (t_0_) visit (OR = 5.24; 95% CI: 3.44–8.05, *p* = 2.2 × 10^−16^). Evaluating the bacterial changes with CT acquisition, we found only 6 genera (including CT itself) being consistently different comparing t_−1_ to t_0_ in cases (Figure 4G). We also observed an increase in the *molBV* score among cases in the transition from before to at the time of CT infection (i.e., comparing t_−1_ and t_0_ visits) (*p* = 0.018, Figure 4H).

BV states mBV-positive and mBV-intermediate were significantly associated with CT infection at the time of CT detection (t_0_) (OR = 3.66, 95% CI: 2.18–6.13, *p* < 0.001, and OR = 2.08, 95% CI: 1.20–3.61, *p* = 0.010, respectively) (see Table 2, cross-sectional (t_0_) model). There was also an elevation in the odds of observing multiple elevated CSTs in the CT case group when using CST-I-A as the reference (Table 2), particularly the BV-associated CSTs dominated by either *Candidatus Lachnocurva vaginae* (CST-IV-A) or *Atopobium vaginae* (CST-IV-B) (OR = 6.50, 95% CI: 2.65–15.92, *p* < 0.001 and OR = 4.31, 95% CI: 2.04–9.12, *p* < 0.001, respectively). Thus, changes in multiple features of the CVM suggest that CT infection has a broad impact on the microbiome composition. There was also a marginally significant increase in the odds of detecting high-risk HPV types (in the mBV model, Table 2) and in the odds of having a higher SBRS (both models, Table 2). School attendance was only significant in the mBV and not the CST model.

### Post-CT-treatment CVM composition (t_+1_ visit)

We evaluated the dynamics of the CVM after antibiotic treatment for CT (t_+1_), primarily with azithromycin or doxycycline. There were no identifiable differences between the CVM in CT cases after treatment compared with the controls at this time (t_+1_) for either bacteria or fungal α- and β-diversity analyses (Figure S5) or specific species using ANCOM. This suggests a resolution to a control-like state at the group level using these measures. However, multivariable modeling indicated that among the cases, the mBV-intermediate group was elevated compared with the controls (OR = 1.83, 95% CI: 1.09–3.08, *p* = 0.02; see Table 2 “post-infection visit” (t_+1_). Similar analysis of CSTs at the post-treatment visit (t_+1_) indicated that CST-III-A and CST-IV-A remained elevated (OR = 2.02, 95% CI: 1.02–4.00, *p* = 0.04 and OR = 2.36, 95% CI: 1.02–5.37, *p* = 0.05, respectively, Table 2). In addition, HR-HPV rates remained significantly elevated among women who had been treated for CT (i.e., CT cases at t_+1_) compared with controls in both models (see Table 2). ANCOM did not identify any significant differences in bacterial species among the case group at t_−1_ vs. t_+1_. The compositional change between the pre- and post-CT CVM of cases was similar to the fluctuation of the CVM over this time (~1 year) in the control group (*p* = 0.2, Figure S6A). Taken together, these data suggest that the case group after treatment reverted to a similar CVM composition that was present prior to CT infection. This reversion is particularly noteworthy given the perturbation of the CVM by CT as measured by the Jensen-Shannon divergence (JSD) difference between t_−1_ and t_0_ compared with the fluctuation of the control group CVM over this similar 6-month period (*p* = 0.00067, Figure S6B). It should be noted that among controls, comparison of the CVM at 6 months (i.e., t_−1_ vs. t_0_) and 12 months (i.e., t_−1_ vs. t_+1_) significantly varied (median [IQR] JSD difference 0.071 [0.23] and 0.10 [0.34], respectively, *p* value = 0.03). This indicates a continuous effect of time on CVM composition even in the absence of a potent perturbation such as infection with CT. In fact, the transition of the CVM from t_−1_ to t_0_ was dependent on the mBV state at the t_−1_ visit. Controls showed balanced transitions across mBV states during this 6-month window, while cases tended to favor a transition to an mBV-positive state (i.e., the likelihood of being in the mBV-positive state at t_0_ was dependent on baseline t_−1_ CVM: greater in mBV-positive, weaker in mBV-intermediate, and weakest (but still stronger than in controls) in mBV-negative (see Figure S6C).

### Post-CT reinfection and sequelae

To examine reinfection rates in participants treated for CT and subsequent incident CT infections in the control group, we leveraged longitudinal data that included clinical testing for CT at each 6-month visit. CT follow-up data beyond the t_+1_ visit was available from 502/560 participants (89.6%) who were representative of the original set of participants (Table S2). Thirty-two (20.6%) of 155 participants with CT infection (i.e., case group) had a CT reinfection compared with 10 (4.1%) of 246 control participants who had a subsequent incident CT infection (OR = 6.11, 95% CI: 2.82–14.42, *p* < 0.001). Among the cases with follow-up, mBV-A was present in 54 at t_−1_ and 57 at t_+1_, with 18 participants having mBV-A at both t_−1_ and t_+1_ (see Venn diagram in Figure S7). To determine the risk that mBV-A contributed to CT reinfection, we utilized a Poisson regression that incorporated the total follow-up time as well as numbers of CT reinfections (Table 3; see model B). Among cases, we observed that the rate of reinfection was increased among participants with mBV-A (adjusted rate ratio [aRR] of 3.58; 95% CI: 1.16–13.28, *p* = 0.034) and among those with mBV-intermediate (aRR = 3.17; 95% CI: 1.18–11.1, *p* = 0.038) (see Table 3; model B).

In addition to CT reinfection, we performed a post hoc exploratory analysis on clinical sequelae of CT infection.^33^ We used self-report of PID and miscarriages. Among the cases, there were 6 participants who had PID, and 15 participants reported a miscarriage. Participants with an mBV-positive CVM at the post-treatment visit (t_+1_) were more likely, albeit not significantly, to develop PID and/or have miscarriages, OR = 2.93 (95% CI: 0.88–11.17, *p* = 0.087) and OR = 4.43 (95% CI: 1.03–30.63, *p* = 0.070), respectively. In terms of CT reinfection and sequelae, we did not observe significant associations with PID (OR = 1.83, 95% CI: 0.14–13.64, *p* = 0.61) or miscarriages (OR = 2.33, 95% CI: 0.29–107.93, *p* = 0.68), but the numbers of events were limited.

## DISCUSSION

This study prospectively examined the natural history of incident CT infection in a large cohort of primarily Black and Hispanic adolescent and young adult females, who have a 5-fold higher risk of CT acquisition compared with their White counterparts but represent only a small fraction of women studied.^4,5^ Cervicovaginal swab samples taken prior to the incident CT infection revealed that participants with mBV had a 62% increased risk for incident CT infection (OR = 1.62, 95% CI: 1.01–2.59, *p* = 0.04). Further categorization of the mBV-positive state integrating CSTs revealed that a specific subtype of mBV containing CST-IV-A was uniquely associated with a risk of CT acquisition (OR = 2.51, 95% CI: 1.40–4.49, *p* = 0.002). Exploring individual bacterial taxa using 10-fold Monte Carlo cross-validation indicated that a group of 10 highly correlated bacteria (e.g., *Candidatus Lachnocurva vaginae*, *Prevotella*, *Megasphaera*, and *Staphylococcus*) collectively increased the risk of CT acquisition. An MRS derived from this set of correlated bacteria indicated the collective risk was greater than the component risk of each bacterium. Cervical samples from the incident visit (t_0_) showed high rates of mBV-positivity with CT and some evidence for co-occurrence of CT with HR-HPVs, although HR-HPV detected at t_−1_ was not a risk factor for CT. At the time of CT infection, we identified a total of 101 bacterial genera that differed between the case-control groups. Analysis of the post-treatment samples indicated that the CVM of CT cases as a group reverted to a compositional state that was nearly identical to the group’s pre-infection CVM. However, there remained a statistically significant elevation of the mBV-intermediate state and elevated levels of CST-IV-A at the post-treatment visit in cases compared with controls. Furthermore, analysis of the post-treatment CVM indicated that the same mBV-positive state (i.e., mBV-A) predicting incident CT infection was also related to the rate of CT reinfection (aRR = 3.58, 95% CI: 1.16–13.28, *p* = 0.034). These data support the observation that a specific set of bacteria are prospectively associated with risk of CT infection.

Our findings demonstrate that various features of the CVM related to BV (including mBV states, CSTs, and specific taxa) are predictive of CT infection and reinfection. BV is characterized by polymicrobialism (i.e., high bacterial diversity) and a lack of *Lactobacillus* dominance.^34^ This is particularly relevant in the context of CT infection, as *Lactobacillus* (especially *L. crispatus*) in the CVM provides protective features by secreting D-lactic acid, which inactivates CT’s infectious particles (elementary bodies) and may mitigate infection.^35^ Our analysis further showed that specific BV features might increase risk beyond what occurs as a result of simply not having a dominance of *Lactobacillus* in the CVM. Identifying these granular BV-associated components may allow for the identification of individuals at risk and offer potential for intervention. This is especially important since some CVM features linked to risk of CT acquisition have also been associated with other health-related conditions. For example, CST-IV-A, which is associated with molecular BV in this study, has been previously linked to persistent BV in a cohort of Kenyan women^36^ and HIV incidence in a separate cohort of African women.^37^ If this holds true across populations, certain subtypes of BV may be more likely to persist and simultaneously increase the risk of sexually transmitted conditions such as CT, as shown in this study, or HIV, as observed in Africa. Incorporating BV stratification using specific marker organisms may therefore be relevant.

In our study, we introduced BV stratification by incorporating CSTs into the mBV definition after observing the association of molecular BV and CST-IV-A (but not CST-IV-B) with incident CT infection. It should be noted that not all CST-IV-A cases are linked to BV, as reported by France et al.^30^ and confirmed in our cohort. In the context of mBV subtypes, mBV-A was characterized by a 33-fold increase in *Candidatus Lachnocurva vaginae* compared with mBV-B. This species was the top taxa associated with CT risk, but its activity was highly correlated with nine other bacteria, suggesting a potential microbial network effect. *Candidatus Lachnocurva vaginae* contains the D-lactate dehydrogenase gene,^38^ which may metabolize D-lactate, potentially weakening D-lactic acid’s protective effect against CT and partially explaining its association with CT risk. While *Candidatus Lachnocurva vaginae* had the strongest individual association with CT infection, the MRS-derived risk bacteria, including *Candidatus Lachnocurva vaginae*, *Prevotella*, and *Acinetobacter* (see Figure 4 for full list), showed a significantly stronger combined association with CT acquisition compared with individual taxa. This suggests an additive effect, potentially indicating that a microbial network, rather than *Candidatus Lachnocurva vaginae* alone, is driving the elevated risk, with *Candidatus Lachnocurva vaginae* possibly serving as a marker rather than a central driver.

The post-treatment CVM analysis identified that mBV-A was also associated with CT reinfection, mirroring its association with the incident CT infection. After CT treatment with antibiotics, women exhibited elevated levels of mBV-intermediate and mBV-A states compared to the controls at the t_+1_ visit. In fact, prior studies have shown that antibiotic treatment alters CSTs as well.^39^ Interestingly, our post-treatment analysis indicated that at the group level, the CVM of cases was similar in overall composition to their baseline CVM. However, individual-level analyses revealed that participants shifted into different BV states. Notably, approximately a third of the original mBV-A participants had this subtype at both t_−1_ and t_+1_, while others transitioned into mBV-A after antibiotic treatment. This allowed us to test whether the mBV-A state was also associated with CT reinfection, which occured in nearly 25% of incident cases, similar to other reports.^22^ Our findings showed that post-treatment mBV-A, mBV-intermediate, and sexual practices measured at the t_+1_ visit were associated with subsequent CT reinfection. Additionally, the CVM profile at the t_+1_ visit indicated an increased risk of PID and miscarriage in cases that had mBV post-CT treatment, although this analysis was limited by small sample size. These findings suggest that mBV may be important in CT infection natural history and its relationship to subsequent sequelae.

This study presents a molecular analysis of the CVM in the context of incident CT infection in a large Black and Hispanic AYA female population who experience the highest rates of CT infection.^4,5^ By leveraging the routine repeat visits of the Mt. Sinai cohort study and novel molecular assessment of BV, we were able to demonstrate that AYA women matched in terms of demographics and behaviors exhibited different risks of CT acquisition based on their CVM. We further show that CVM features associated with incident CT infection were also associated with subsequent reinfection. This understanding may form the basis of new public health measures to reduce the burden of CT infections by development of preventive and therapeutic strategies based on more granular features of the CVM.

### Limitations of the study

Limitations of the study include the sampling interval between the incident CT infection and the pre-infection visit. Although we used samples taken 6 months prior to the incident CT infection, the interval may still not be completely representative of the true CVM state at the time of CT exposure. In addition, we cannot disentangle the effects of the antibiotics from the effects of CT elimination on the resultant CVM since all participants with CT were treated with antibiotics. Due to sample size considerations, CST subgroup analyses were limited. That is, while most CSTs had adequate sample size, we did not detect CST-II, and few individuals had CST-III-B. Additionally, the post hoc analysis of clinically relevant outcomes, including PID and miscarriage, while showing intriguing associations should be considered exploratory, as we were limited to self-reported data and small sample sizes.

## RESOURCE AVAILABILITY

### Lead contact

Requests for further information and resources should be directed to and will be fulfilled by the lead contact, Robert D. Burk (robert.burk@einsteinmed.edu).

### Materials availability

This study did not generate new, unique reagents.

### Data and code availability

Full 16SV4 rRNA and ITS1 sequence data for the Mt. Sinai CT cohort have been uploaded and are available for download in Qiita study ID: 14884.

## STAR★METHODS

### EXPERIMENTAL MODEL AND STUDY PARTICIPANT DETAILS

Participants were selected from a longitudinal dynamic cohort study of sexually active adolescent female patients receiving gynecological care at Mount Sinai Adolescent Health Center (MSAHC) in New York City. Overall population characteristics, eligibility criteria (which included female sex at birth and history of sexual intercourse), and study design of the larger cohort have been previously described.^40^ Briefly, study participants were enrolled between the ages of 13–21 and received a gynecological examination at each study visit, approximately every 6 months. Routine screening for gonorrhea and chlamydia was performed at each visit and as clinically indicated using GEN-PROBE APTIMA (Hologic, Marlborough, MA) assays performed by the clinical laboratory. Clinical indications for testing included patient reports of a positive exposure (i.e., partner with a known STI), or a patient’s own symptoms (e.g., vaginal discharge, itch, pelvic pain, and/or bleeding with intercourse). In addition, patients could request to test whether the CT infection was resolved, which was usually done within 4 weeks after treatment. Research questionnaires were administered at every study visit, which included questions on age, sex, race, ethnicity, place of birth, sexual behaviors, sexual partners, history of STIs, condom use, drug and alcohol use, and other related factors.^26^ All participants were of female sex per eligibility criteria for the study, and gender identity was not collected for analysis purposes. Written informed consent was obtained from all participants prior to enrollment. The Institutional Review Board at Ichan School of Medicine at Mount Sinai and Mount Sinai Hospitals Group approved the study (Federalwide Assurance # FWA00005651).

#### Nested case-control study design

We used a nested case-control study design to select cases with incident CT detected after enrollment into the cohort, i.e., CT negative at a t_−1_ and positive at t_0_ visit. Participant samples were selected as part of a prospective open cohort, which began enrollment in October 2007 and continued through this analysis using all data until October 2022. Thus, enrollment and follow-up occurred during this interval. Two control individuals (CT negative at t_−1_ and t_0_) were then matched to each case on age (±3 years), year of enrollment, and prior history of CT. Controls were allowed to become cases if they became CT positive later in the study.

Banked DNA samples for analysis of the CVM were obtained from specimens collected at the cohort study visits using a cervical Cytobrush^®^ placed in PreservCyt transport medium (ThinPrep^®^; Hologic) following the same procedure as for Pap smears. Up to three cervicovaginal samples from each participant were tested for the CVM (see Figure 1 for study design). In a nested case-control design, cases can act as controls and vice versa (e.g., at the t_−1_ and t_+1_ visits). Additionally our participants are matched on person-time, come from the same population, which helps reduce selection bias and improve the comparability between groups.

A total of 187 participants were identified with an incident CT infection during the cohort study follow-up at the time of selection (January 2020) and were matched to 373 controls (one case had only a single matched control available for inclusion). Cervicovaginal samples collected prior to the infection (t_−1_) were available for all 187 cases with a median (interquartile range, IQR) time prior to incident CT detection (t_0_) of 6.75 (3.35) months, with a comparable sample available from 373 controls. Samples tested at the visit after CT detection and matched visit for controls were collected 6.70 (1.97) and 6.83 (2.83) months after t_0_ for cases and controls, respectively.

### METHOD DETAILS

Cervicovaginal samples were stored immediately at −20°C until transport to the research lab at the Albert Einstein College of Medicine. The samples were transferred to a 15 ml tube in the lab and gently centrifuged at 1500 RPM for 5 min. After removing the supernatant by decanting, the pellets were rinsed in 3 ml of TE (10 mM Tris, 1.0 mM EDTA). This solution was then vortexed and centrifuged at 1500 RPM for 5 min and the supernatant was removed by decanting. The remaining pellet and leftover solution (~150 μl) were used for DNA isolation via column processing with the QIAamp Mini spin column (Qiagen, Valencia, CA) following the manufacturer’s protocol. The purified DNA was eluted in 150 μl of elution buffer (10mM Tris/0.5mM EDTA, pH 9) and used initially for HPV DNA analyses and then stored at −20°C in a non-frost-free freezer.

PCR for bacterial communities was performed using forward (515F) GTGYCAGCMGCCGCGGTA and reverse (806R) GGACTACHVGGGTWTCTAAT primers that amplify the V4 hypervariable region of the prokaryotic 16S rRNA gene.^41,42^ All primers contained unique Golay barcodes to allow for dual indexing of each sample and were purchased from IDT (IDT, Coralville, IA). PCRs were conducted in a 25 μl reaction with 2 μl input of template DNA, 16.75 μl of ddH20, 2.5 μl of Platinum 10X PCR buffer (Invitrogen, Waltham, MA), 0.75 μl of MgCl_2_ (50 mM, Invitrogen), 0.5 μl of dNTP mix (10mM each, Roche, Basel, Switzerland), 0.25 μl AmpliTaq Gold, polymerase (5 U/μl, Applied Biosystems, Carlsbad, CA), 0.25 μl of Platinum Taq DNA Polymerase (10 U/μl, Invitrogen), and 1 μl (5 μM) of each primer. Thermocycling conditions included an initial denaturation at 95°C for 5 min, followed by 15 cycles of 95°C for 1 min, 55°C for 1 min, 72°C for 1 min, followed by 15 cycles of 95°C for 1 min, 60°C for 1 min, 72°C for 1 min, and a final extension at 72°C for 10 min.

In order to more comprehensively profile the CVM, we additionally performed ITS1 sequencing, which identifies fungal and eukaryotic species. These organisms have been reported to modulate the CVM despite their relatively minor biomass as compared to bacteria.^43^ PCR for eukaryotic communities was performed using barcoded forward (48F) ACACACCGCCCGTCGCTACT and reverse (217R) TTTCGCTGCGTTCTTCATCG primers (IDT) that amplify the ITS1 region of the prokaryotic ribosomal gene cluster.^44,45^ PCRs were conducted in a 25 μl reaction with 10 μl input of template DNA, 8.75 μl of ddH20, 2.5 μl of Platinum 10x PCR buffer (Invitrogen), 0.75 μl of MgCl_2_ (50 mM, Invitrogen), 0.5 μl of dNTP mix (10 mM each, Roche), 0.25 μl AmpliTaq Gold polymerase (5 U/μl, Applied Biosystems), 0.25 μl of Platinum Taq DNA Polymerase (10 U/μl, Invitrogen), and 1 μl (5 μM) of each primer (IDT, Coralville, IA). Thermocycling conditions included an initial denaturation at 95°C for 5 min, followed by 35 cycles of 95°C for 30 s, 55°C for 30 s, 72°C for 2 min, followed by a final extension at 72°C for 10 min. All PCRs were conducted in a Veriti Thermal Cycler (Applied Biosystems, Foster City, CA) and PCR products were verified by gel electrophoresis.

PCR products for each sample were pooled by PCR assay (16S and ITS1) in approximately equal concentrations and 100 μl of the pooled products were loaded into a 3% agarose gel and run at 80V for 3 h to separate the DNA fragments. The DNA fragment for each assay was excised and purified with a QIAquick Gel Extraction Kit (Qiagen) and quantified using a Qubit High Sensitivity dsDNA assay (Invitrogen). NGS library preparation was conducted on the purified pooled PCR products from each assay with a KAPA LTP Library Preparation Kit (KAPA Biosystems, Wilmington, MA) according to the manufacturer’s protocol. The library amplicons were validated on a 2100 Bioanalyzer (Agilent Technologies, Santa Clara, CA) and sequencing of libraries was carried out on an Illumina NovaSeq 6000 using the 2×250 bp paired-end reads kit.

### QUANTIFICATION AND STATISTICAL ANALYSIS

Sequence reads were clustered into amplicon sequence variants (ASVs) using DADA2 and taxonomy was assigned using a custom cervicovaginal microbiome specific database^27^ employing a Naive Bayesian classifier. We assessed the presence of bacterial vaginosis (BV) using a previously validated molecular score from the 16SV4 reads (*molBV*). The *molBV* score converts 16S rRNA gene amplicon sequences into a Nugent-like score from 1–10.^27^ This assay provides a measure of the cervicovaginal microbiome from DNA isolated from standard cytology/Pap-smear samples. It is particularly useful when measurements of BV are not available, but cervicovaginal DNA is available (e.g., from HPV testing). Cervicovaginal samples with *molBV* scores of 1–3 were classified as mBV-negative, those with >3 - <7 were classified as mBV-intermediate, and those with 7–10 were classified as mBV-positive. mBV states determined by *molBV* were robust across three large independent cohorts spanning US and continental African women as demonstrated by AUCs of 0.88–0.98.^27^ Cervicovaginal microbiome community state types (CSTs) were generated using VALENCIA.^30^ To incorporate CST definitions into our mBV analysis we dichotomized mBV-positive (i.e., *molBV* 7–10) participants into mBV-A subtype if they concurrently had CST-IVA and into mBV-B if they had *molBV* 7–10 and not CST-IVA.

We performed a sensitivity analysis comparing *molBV* categorical states (i.e., mBV-positive, mBV-intermediate vs. mBV-negative), the clinical Amsel BV diagnosis from the participant clinical visit, and CST distributions (Figure S8; Table S3). The Amsel diagnosis and CST distributions concurred with the *molBV* derived mBV-positive and mBV-intermediate categories. Comparison of the models using Akaike Information Criterion (AIC) and Schwarz’s Bayesian Information Criterion (BIC), which measure goodness of fit, indicated that the mBV models had the best fit of the data.

NGS reads from this study were deposited in the Qiita repository^46^ (Study ID: 14884).

Negative controls (*n* = 17) and positive controls (*n* = 17) were used to control for contamination and downstream amplification, sequencing, and bioinformatics. The negative controls had an average (SD) read recovery of 2,803 (1,000) reads compared with the true samples which had an average of 36,384 (13,512) reads, (*p* value < 0.001). For ITS1 sequencing, the recovery was 1,741 (4,563) reads in the negative controls and 7,774 (20,879) in true samples (*p* < 0.001). The negative control was water. The positive control was purchased from ZymoBIOMICS Microbial DNA Standard (Zymo Research Corp., Irvine, CA) and after amplification and sequencing showed the expected composition including the bacteria-*Pseudomonas aeruginosa*, *Escherichia coli*, *Salmonella enterica*, *Lactobacillus fermentum*, *Enterococcus faecalis*, *Staphylococcus aureus*, *Listeria monocytogenes* and *Bacillus subtilis*, and fungi-*Saccharomyces cerevisiae* and *Cryptococcus neoformans*.

Conditional logistic regression models were fitted using the *survival* package in R to assess the associations between categorical *mBV* state (*mBV*-intermediate and *mBV*-positive vs. *mBV*-negative) and CT at index visit (t_0_) and prospectively (using the CVM assessed at study visit (t_−1_), approximately 6 months prior to detection of CT in cases). The odds ratios (ORs) of incident CT based on *m*BV states were estimated by comparing subjects who were *mBV*-positive and *mBV*-intermediate to *mBV*-negative subjects, adjusting for current school attendance, presence of more than one oncogenic HR-HPV type (i.e., HPV16, 18, 31, 33, 35, 39, 45, 51, 52, 56, 58 and 59, measured by allele-specific oligonucleotide hybridization as described^47^) and sexual risk behavior scores.^28^ All confounding variables for the effect between incident CT and CVM were measured at the time of CVM sampling (i.e., at t_−1_, t_0_, and t_+1_). The continuous sexual risk behavior composite score is a linear combination of the following variables with categorical states shown in parentheses: lifetime vaginal sex partners (1–4, with 4 representing 4 or more partners), recent (past six months) number of sex partners (0–2, with 2 representing 2 or more partners), history of any pregnancy (0–1), emergency contraception use (0–1), condom usage during recent sex (0–1, with 1 representing never or rarely), ever anal sex (0–1), and lifetime number of anal sex partners (0–2, with 2 representing 2 or more partners); higher values indicate higher sexual risk behavior. In terms of microbial measures, α-diversity (i.e., Chao1 and Shannon indices) and β-diversity (Jensen Shannon divergence (JSD) distance) were calculated using the *phyloseq* package^48^ in R. Statistical significance in α-diversity was determined using the Wilcoxon rank sum test using a core function R, while PERMANOVA was used to assess significance and obtain R^2^ in β-diversity with the *vegan* R package.^49^ Alluvial plots, representing mBV state transitions between t_−1_ and t_0_ visits were constructed using the ggplot2 package and the geom_alluvium function. For cross-validation, data was randomly split (50:50) between testing and training sets with effects representing the pooled combined effects of replicable taxa across the 10 testing folds (see Figure S4 for all cross-validation results). Biomarker discovery was performed using ANCOM,^50^ a tool designed specifically for identification of differential bacterial taxa in the context of compositional microbiome data using regression, in the testing set and effect estimates for biomarkers significant after adjustment for FDR < 0.05 were modeled in the testing folds with final mixed-effect pooled effects reported.

To evaluate cross-validated bacterial taxa from the t_−1_ visit, Pearson correlation coefficients were calculated using the R *stats* package. Determination of a microbial risk score (MRS),^32^ an approach analogous to polygenic risk score, combines measures of multiple bacterial into a single value. We calculated the diversity MRS (i.e., MRSa) by performing the corresponding α-diversity calculations (i.e., Chao1 and Shannon) using the *phyloseq* package on the subset of bacterial taxa found to be significantly different between cases (i.e., those that acquired CT) vs. controls using ANCOM followed by cross-validation. To calculate the abundance based MRS (i.e., MRSs) we summed the cross-validated bacterial taxa and applied a weighted sum (weight based on OR of taxon with respect to CT acquisition) as described in the original MRS paper.^32^ The purpose of the score is to measure the effect of a given bacterial network. For evaluation of subsequent CT reinfection rates after the t_+1_ visit, we used the time to each CT reinfection and Poisson regression within the glm function of the *stats* package with adjustment using the same variables used in the core analysis (i.e., SRBS, HR-HPV positivity, and school attendance).

In addition to CT reinfection, we performed a post-hoc exploratory analysis on clinical sequelae of CT infection using self-reported incidence of PID and miscarriages. Regarding self-reported PID, participants were asked on the study questionnaire at each follow-up visit if “During the past 6 months, have you been told by a doctor or health care provider that you have Pelvic Inflammatory Disease (PID)”. This was reported as yes/no/don’t know. Information on prior pregnancies was recorded from clinical interviews conducted during the physical examinations at each study visit and included information on spontaneous abortions. This was recorded as the number of pregnancies that resulted in spontaneous abortion since the last study visit.

#### Mediation analysis

Although sexual behavior was not found to be statistically associated with incident *Chlamydia trachomatis* (CT) infection in this cohort, it is considered an important risk factor for not only CT infection but also development of BV. To analyze this in greater detail, we performed a mediation analysis as shown below.


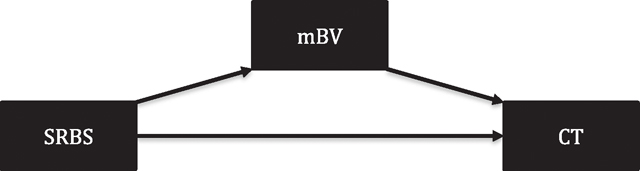


We employed the mediate function from the *mediation* package in R. This analysis was conducted with 1000 bootstrap simulations to estimate the Average Causal Mediation Effect (ACME), Average Direct Effect (ADE), and the proportion of the total effect that is mediated. For each mediation analysis, we created binary dummy variables for the BV categories (mBV-Negative, mBV-Intermediate, and mBV-Positive) and modeled the mediator (mBV) and outcome (incident CT infection) using logistic regression. The mediator models included high-risk HPV status and school attendance as covariates. This approach allowed us to quantify the direct and indirect effects of SRBS on CT infection, mediated through BV. The results are presented below.

**Table:** 

Value	Estimate	95% CI Lower	95% CI Upper	*p* value

ACME	0.00021	−0.0016	0.000	0.82
ADE	−0.00053	−0.025	0.020	0.93
Prop. Mediated	−0.67	−0.91	0.750	0.97
Total Effect	−0.00032	−0.025	0.020	0.94

The direct effect of SRBS on CT infection, not mediated by BV, was negligible and not statistically significant (ADE value). The combined direct and indirect effects of SRBS on CT infection were similarly negligible and not statistically significant (total effect). Finally, the proportion of the total effect that is mediated by BV was not significant. These results might be surprising if taken outside the context of the study cohort characteristics, which included sexually active AYAs, evident in the case-control comparisons of sexual behavior (see Table 1 and Table S1). The characteristics of the study cohort allowed us to directly measure the effect of BV on CT while limiting the impact of sexual behavior. This result is important as it provides additional support that sexual behavior was not a factor driving the association with our main exposure (i.e., the CVM), which is important because of the association of sex with both BV and CT.

## Supplementary Material

Document S1. Tables S1–S3

2

## Figures and Tables

**Table 1.** Characteristics of participants by visit

	Pre-infection visit (t_−1_), *n* = 560			Cross-sectional visit (t_0_), *n* = 560			Post-infection visit (t_+1_), *n* = 503		

Variables	CT cases	CT controls	*p* value	CT cases	CT controls	*p* value	CT cases	CT controls	*p* value

*n*	187	373	N/A	187	373	N/A	162	342	N/A
Age mean ± SD (years)	19.7 ± 2.32	19.72 ± 2.12	0.91	20.43 ± 2.31	20.44 ± 2.1	0.86	20.94 ± 2.21	21.12 ± 2.08	0.42
mBV-categorical									
mBV-negative^a^	41 (22.8%)	115 (32.2%)	N/A	30 (16.5%)	124 (33.8%)	N/A	38 (23.8%)	98 (29.3%)	N/A
mBV-intermediate	67 (37.2%)	123 (34.5%)	0.081	60 (33%)	134 (36.5%)	0.019	64 (40%)	111 (33.1%)	0.12
mBV-positive	72 (40%)	119 (33.3%)	0.029	92 (50.5%)	109 (29.7%)	<0.0001	58 (36.2%)	126 (37.6%)	0.54
Missing^b^	7 (3.7%)	16 (4.3%)	N/A	5 (2.7%)	6 (1.6%)	N/A	2 (1.2%)	7 (2.0%)	N/A
CSTs									
CST-I^a^	38 (21.1%)	103 (28.9%)	N/A	16 (8.7%)	106 (28.8%)	N/A	25 (15.6%)	73 (21.7%)	N/A
CST-III-A	43 (23.9%)	100 (28%)	0.60	46 (25.1%)	91 (24.7%)	1.30E-04	54 (33.8%)	98 (29.2%)	0.13
CST-III-B	5 (2.8%)	13 (3.6%)	1.00	9 (4.9%)	24 (6.5%)	0.063	5 (3.1%)	19 (5.7%)	0.79
CST-IV-A	32 (17.8%)	36 (10.1%)	0.005	38 (20.8%)	51 (13.9%)	2.22E-06	26 (16.2%)	46 (13.7%)	0.18
CST-IV-B	62 (34.4%)	105 (29.4%)	0.067	73 (40.4%)	95 (26.1%)	1.94E-08	50 (31.2%)	99 (29.8%)	0.21
Missing^b^	7 (3.7%)	16 (4.3%)	N/A	5 (2.7%)	6 (1.6%)	N/A	2 (1.2%)	7 (2.0%)	N/A
Sexual risk behavior score^c^, mean ± SD	5.71 ± 1.68	5.71 ± 1.89	0.89	6.14 ± 1.48	5.77 ± 1.87	0.07	6.16 ± 1.47	5.98 ± 1.72	0.36
High-risk HPV									
Positive	4 (2.1%)	12 (3.2%)	N/A	8 (4.3%)	6 (1.6%)	N/A	11 (6.8%)	9 (2.6%)	N/A
Negative	183 (97.9%)	358 (96.8%)	0.60	179 (95.7%)	367 (98.4%)	0.082	151 (93.2%)	333 (97.4%)	0.047
Currently attending school									
Yes	137 (74.1%)	277 (74.5%)	N/A	130 (69.5%)	239 (64.1%)	N/A	106 (65.4%)	201 (58.8%)	N/A
No	48 (25.9%)	95 (25.5%)	0.92	57 (30.5%)	134 (35.9%)	0.22	56 (34.6%)	141 (41.2%)	0.17
Missing	2 (1.1%)	1 (0.3%)	N/A	0 (0.0%)	0 (0.0%)	N/A	0 (0.0%)	0 (0.0%)	N/A
Race/ethnicity									
African-American/not Hispanic	80 (42.8%)	133(35.7%)	N/A	80 (42.8%)	133(35.7%)	N/A	71 (43.8%)	124 (36.3%)	N/A
African-American/Hispanic	32 (17.1%)	49 (13.1%)	0.789	32 (17.1%)	49 (13.1%)	0.789	28 (17.3%)	45 (13.2%)	0.778
No reported race/Hispanic	65 (34.8%)	145 (38.9%)	0.183	65 (34.8%)	145 (38.9%)	0.183	54 (33.3%)	135 (39.5%)	0.104
Other race/not Hispanic	2 (1.1%)	19(5.1%)	0.008	2 (1.1%)	19(5.1%)	0.008	2 (1.2%)	15 (4.4%)	0.059
Unknown race	8 (4.3%)	27 (7.2%)	0.126	8 (4.3%)	27 (7.2%)	0.126	7 (4.3%)	23 (6.7%)	0.216

Continuous value significance assessed using non-parametric Wilcoxon rank sum test. Categorical variable significance assessed using Fisher test. mBV, molecular bacterial vaginosis based on *molBV* scores (see STAR Methods); CSTs, community state types; high-risk HPV types: HPV16, −18, −31, −33, −35, −39, −45, −51, −52, −56, −58, and −59.

a mBV-negative and CST-I are used as the reference groups for Fisher test.

b mBV and CSTs are missing for participants who had 16S sequencing depth <10,000 reads.

c Sexual risk behavior score is a composite linear measure that incorporates the total number of lifetime vaginal sexual partners, number of vaginal partners in the last 6 months, lifetime number of pregnancies, condom usage during recent sex, anal sex history, and history of emergency contraceptive use (higher values indicating higher risk behavior; see methods for full score description and Table S1 for comparison of individual behavior variables).

**Table 2.** Analysis of incident *Chlamydia trachomatis* infection-associated factors at the pre-, incident, and post-infection visit

Covariate	aOR^a^ (95% CI)	*p* value	aOR^a^ (95% CI)	*p* value	aOR^a^ (95% CI)	*p* value

	Pre-infection visit (t_−1_), *n* = 560		Cross-sectional visit (t_0_), *n* = 560		Post-infection visit (t_+1_), *n* = 503	

mBV (ref: mBV-negative)						
mBV-intermediate	1.46 (0.90–2.38)	0.12	2.08 (1.20–3.61)	0.01	1.83 (1.09–3.08)	0.02
mBV-positive	1.62 (1.01–2.59)	0.04	3.66 (2.18–6.13)	<0.001	1.26 (0.73–2.18)	0.41
Sexual risk behavior score^b^	1.00 (0.89–1.11)	0.95	1.13 (1.00–1.29)	0.05	1.14 (1.00–1.3)	0.04
High-risk HPV (ref: negative)						
High-risk HPV-positive	0.62 (0.20–1.94)	0.41	2.95 (0.98–8.88)	0.05	3.01 (1.18–7.70)	0.02
Currently attending school (ref: no)						
Yes	0.94 (0.59–1.51)	0.81	1.78 (1.12–2.84)	0.01	1.37 (0.87–2.16)	0.17

	Pre-infection visit (t_−1_)		Cross-sectional visit (t_0_)		Post-infection visit (t_+1_)	

CST (ref: CST-I-A)						
CST-III-A	1.1 (0.58–2.08)	0.77	2.83 (1.3–6.16)	0.010	2.02 (1.02–4.00)	0.04
CST-III-B	1.15 (0.32–4.12)	0.83	2.58 (0.83–8.06)	0.10	1.18 (0.34–4.1)	0.79
CST-IV-A	2.46 (1.16–5.19)	0.02	6.50 (2.65–15.92)	<0.001	2.36 (1.02–5.47)	0.05
CST-IV-B	1.44 (0.77–2.7)	0.25	4.31 (2.04–9.12)	<0.001	1.74 (0.85–3.58)	0.13
Sexual risk behavior score^b^	0.97 (0.85–1.11)	0.63	1.16 (1.00–1.34)	0.05	1.12 (0.97–1.29)	0.12
High-risk HPV (ref: negative)						
High-risk HPV-positive	0.66 (0.17–2.53)	0.55	1.6 (0.44–5.91)	0.48	2.96 (1.02–8.64)	0.05
Currently attending school (ref: no)						
Yes	0.97 (0.54–1.75)	0.92	1.39 (0.81–2.36)	0.23	1.48 (0.91–2.42)	0.12

Post-infection (t_+1_) visit models the outcome of the CVM measured at t_+1_ following treatment of CT with antibiotics with respect to the matched controls using the original risk-set sampling designation at t_0_. See also Figures S1 and S2.

Abbreviations as described in Table 1.

a Adjusted odds ratio (aOR) based on multivariable conditional logistic regression.

b OR is per unit increase in score.

**Table 3.** Stratified molecular BV states and risk of CT incident infection and reinfection

	(A) Pre-infection visit (t_−1_) CVM and incident infection at t_0_		(B) Post-infection visit (t_+1_) CVM and CT reinfection at t_0_	

Covariate	aOR^a^ (95% CI), *n* = 560	*p* value	aRR (95% CI),^b^ *n* = 160	*p* value

mBV (ref: mBV-negative)				
mBV-intermediate	1.41 (0.85–2.33)	0.18	3.17 (1.18–11.06)	0.038
*m*BV-A	2.38 (1.21–4.69)	0.012	3.58 (1.16–13.28)	0.034
*m*BV-B	1.46 (0.85–2.51)	0.17	1.57 (0.44–6.32)	0.49
Sexual risk behavior score^c^	0.98 (0.87–1.1)	0.72	1.33 (1.04–1.74)	0.030
High-risk HPV (ref: negative)				
High-risk HPV-positive	0.51 (0.14–1.88)	0.31	1.6 (0.46–4.37)	0.40
Currently attending school (ref: no)				
Yes	0.79 (0.48–1.3)	0.36	1.39 (0.68–3.06)	0.39
Time to CT^d^ (ref: timed)				
Time1	0.77 (0.45–1.32)	0.35	N/A	N/A
Time2	1.34 (0.82–2.2)	0.25	N/A	N/A

mBV-A, mBV-positive, and CST-IV-A positive; mBV-B, mBV-positive, and CST-IV-A negative. Abbreviations as described in Tables 1 and 2. See also Figures S1, S5, S6, and S7.

a Adjusted odds ratio (aOR) based on multivariable conditional logistic regression with incident CT status at visit t_0_ as the outcome.

b Adjusted rate ratio (aRR), calculated by multivariable Poisson regression with post-t_+1_ CT infection counts as the outcome.

c Sexual risk behavior score as described in Table 1.

d Time variable was treated as a piecewise linear model with two knots at 6.3 and 7.5 months (<6.3 months, 6.3–7.5 months, and >7.5 months).

**KEY RESOURCES TABLE:** 

REAGENT or RESOURCE	SOURCE	IDENTIFIER
Chemicals, peptides, and recombinant proteins		
QIAamp Mini spin column	Qiagen, Valencia, CA	51306
Platinum 10XPCR buffer	Invitrogen, Waltham, MA	10966018
MgCl_2_	Applied Biosystems, Carlsbad, CA	4311806
dNTP mix	Roche, Basel, Switzerland	11814362001
AmpliTaq Gold, polymerase	Applied Biosystems, Carlsbad, CA	4311806
Platinum Taq DNA Polymerase	Invitrogen, Waltham, MA	10966018
Deposited data		
16SV4 rRNA & ITS1 sequencing reads	Qiita	Qiita Repository Study ID: 14884
Software and algorithms		
*molBV*	github	github.com/musyk07/molBV
VALENCIA	github	github.com/ravel-lab/VALENCIA

## Highlights

- Longitudinal design to study CVM’s impact on CT incidence, reinfection, and sequelae
- Molecular BV subtype with CST-IV-A identified as a key risk for incident CT infection
- Described a network of ten bacterial genera associated with incident CT
- Molecular BV subtype with CST-IV-A elevates the risk of CT reinfection post treatment
