# Supplementary material for: Cervicovaginal microbiome and natural history of Chlamydia trachomatis in adolescents and young women
Source: Cell. Author manuscript; Available in PMC 2025 Apr 28. (PMC12035847; doi:10.1016/j.cell.2024.12.011)
Supplement: Document S1. Tables S1–S3 [file NIHMS2071029-supplement-Document_S1__Tables_S1_S3.pdf]

**Cell, Volume *188***

# Supplemental information

# Cervicovaginal microbiome and natural history of *Chlamydia trachomatis* in adolescents and young women

**Mykhaylo Usyk, Luke Carlson, Nicolas F. Schlecht, Christopher C. Sollecito, Evan Grassi, Fanua Wiek, Shankar Viswanathan, Howard D. Strickler, Anne Nucci-Sack, Angela Diaz, and Robert D. Burk**

## Supplemental Tables

**Supplemental Table 1: Sexual Risk Behavior Score (SRBS) at $t_0$, related to SRBS scores shown in Tables 1, 2 and 3**

| Variables | Cases | Controls | p-value |
|---|---|---|---|
| Number of participants | 187 | 373 | |
| SRBS, Mean± SD | 5.71± 1.68 | 5.71± 1.89 | 0.89 |
| Lifetime vaginal sex partners, Mean± SD | 3.22± 0.96 | 3.06± 1.07 | 0.14 |
| Past 6 months vaginal sex partners, Mean± SD | 1.39± 0.53 | 1.33± 0.54 | 0.22 |
| Lifetime number of pregnancies, Mean± SD | 0.11± 0.32 | 0.16± 0.36 | 0.21 |
| Condom usage | | | |
| Never | 30 (17.2%) | 84 (22.6%) | - |
| Rarely | 28 (15.1%) | 71 (19.1%) | 0.99 |
| Sometimes | 42 (22.6%) | 76 (20.4%) | 0.21 |
| Most Times | 39 (21.0%) | 63 (16.9%) | 0.11 |
| All Time | 39 (21.0%) | 58 (15.6%) | 0.06 |
| Doesn't Apply | 6 (3.2%) | 20 (5.4%) | 0.81 |
| Missing (n = 2) | 1 (0.5%) | 1 (0.3%) | 0.48 |
| Ever emergency contraceptive | | | |
| No | 39 (29.5%) | 61 (22.7%) | 0.14 |
| Yes | 93 (70.5%) | 208 (77.3%) | - |
| Missing (n = 159) | 55 (29.4%) | 104 (27.9%) | 0.46 |
| Ever Anal sex | | | |
| No | 65 (59.6%) | 133 (57.6%) | 0.81 |
| Yes | 44 (40.4%) | 98 (42.4%) | - |
| Missing (n = 220) | 78 (41.7%) | 142 (38.1%) | 0.61 |

Table shows the composite sexual risk behavior score (SRBS, line 2) and the component elements broken down. SRBS is a linear summation of the components shown, except for condom use, which is reversed in rank order as described in Gradissimo et al.[28]

**Supplemental Table 2. Comparison of Total Cohort and Cohort with Follow-up Data, related to Table 3 model (B) (i.e. characteristics of patients with follow-up data vs. full cohort)**

| Variable | Total Group ($t_0$) | Group with Follow-up ($t_0$) | p-value |
|---|---|---|---|
| Number of participants | 560 | 502 | |
| Case-Control | | | |
| Cases | 187 (33.4%) | 160 (31.9%) | 0.60 |
| Controls | 373 (66.6%) | 342 (68.1%) | - |
| Age, Mean ± SD (years) | 20.44± 2.17 | 20.39± 2.16 | 0.77 |
| mBV at $t_0$ | | | |
| mBV-Negative | 154 (28.1%) | 133 (27.1%) | 0.14 |
| mBV-Intermediate | 194 (35.3%) | 174 (35.4%) | - |
| mBV-Positive | 201 (36.6%) | 184 (37.5%) | - |
| Missing (n = 11) | | | |
| Sexual Risk Behavior Score, Mean ± SD | 5.89 ± 1.76 | 5.88 ± 1.74 | 0.90 |
| High-Risk HPV-Positive | | | |
| negative | 546 (97.5%) | 488 (97.2%) | 0.85 |
| positive | 14 (2.5%) | 14 (2.8%) | - |
| Currently attending school | | | |
| No | 191 (34.1%) | 170 (33.9%) | 0.95 |
| Yes | 369 (65.9%) | 332 (66.1%) | - |
| See table 1 for abbreviations. | | | |

**Supplemental Table 3: Sensitivity Analysis of Incident *Chlamydia trachomatis* Infection Associated Factors at the Incident Visit comparing three approaches for analyzing the CVM, related to Figure 2 and STAR Methods**

| Model | Variable | coef | se(coef) | p-value |
|---|---|---|---|---|
| **mBV[1]** | | | | |
| | mBV-Intermediate | 0.73 | 0.27 | 0.0087 |
| | mBV-Positive | 1.29 | 0.26 | 8.32E-07 |
| | SRBS^ | 0.12 | 0.064 | 0.049 |
| | High-Risk HPV | 1.08 | 0.56 | 0.054 |
| | Currently Attending School | 0.57 | 0.23 | 0.014 |
| **Amsel[2]** | | | | |
| | Amsel-Inconclusive | 0.24 | 0.63 | 0.70 |
| | Amsel-BV | 1.09 | 0.39 | 0.0052 |
| | SRBS^ | 0.14 | 0.069 | 0.037 |
| | High-Risk HPV | 0.60 | 0.63 | 0.33 |
| | Currently Attending School | 0.31 | 0.25 | 0.22 |
| **CST[3]** | | | | |
| | CST.III | 0.97 | 0.33 | 0.0037 |
| | CST.IV | 1.67 | 0.34 | 1.43E-06 |
| | SRBS^ | 0.12 | 0.062 | 0.039 |
| | High-Risk HPV | 0.96 | 0.55 | 0.083 |
| | Currently Attending School | 0.40 | 0.22 | 0.068 |

Table shows the results of a sensitivity analysis comparing three approaches for analyzing the cervicovaginal microbiome. All three models use incident CT as the outcome while holding SRBS, HR-HPV status, and school attendance constant and varying the CVM/BV measurements at $t_0$ (i.e., mBV, Amsel, and CSTs) to test consistency across these approaches. See Table 1 for abbreviations.

Model selection statistics using Akaike Information Criterion (AIC) and Schwarz's Bayesian Information Criterion (BIC) are shown below for each model (lower value indicates better model fit).

1. mBV AIC and BIC values are 365.16 and 381.18, respectively.
2. Amsel's AIC and BIC values are 397.81 and 413.96, respectively.
3. CST AIC and BIC values are 375.49 and 391.64, respectively.
