# Supplementary material for: Cervicovaginal microbiome and natural history of Chlamydia trachomatis in adolescents and young women
Source: Cell. Author manuscript; Available in PMC 2025 Apr 28. (PMC12035847; doi:10.1016/j.cell.2024.12.011)
Supplement: 2 [file NIHMS2071029-supplement-2.pdf]

CellPress
OPEN ACCESS

Supplemental figures

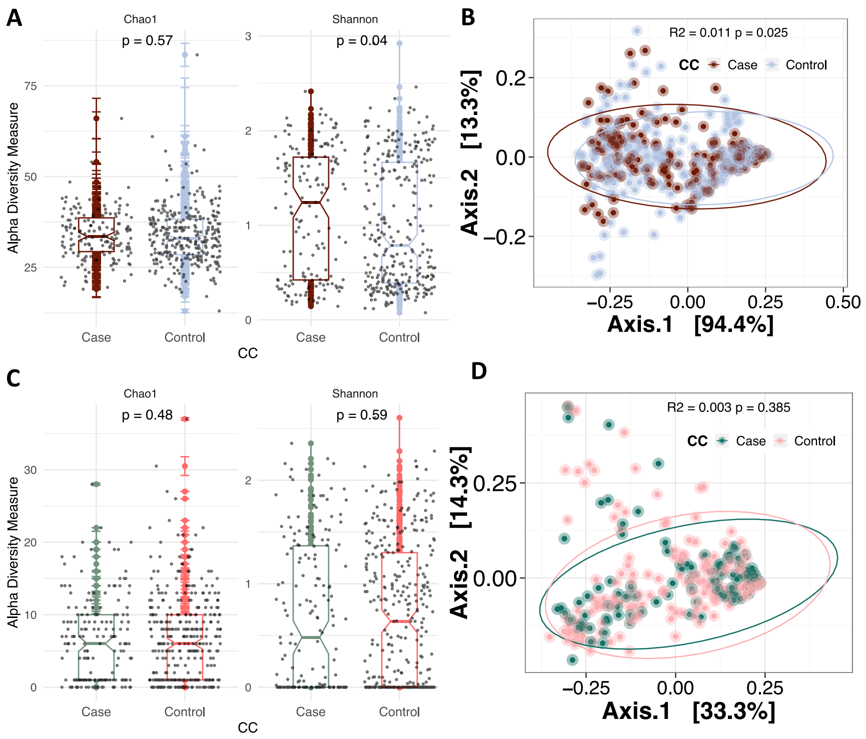

Figure S1. CVM diversity at the visit prior to incident CT infection (visit t_{-1}), related to Figure 2 and Tables 2 and 3

Figure shows the α and β diversity of the CVM at the pre-infection visit (i.e., t_{-1}, prior to incident CT infection).

(A and C) The α diversity of the bacterial and fungal components of the CVM, respectively. For (A) and (C), *p* values are based on Wilcoxon rank sum test (note that the y axis scales are different).

(B and D) β diversity calculated using principal coordinate analysis (PCoA) plots. These measure the overall community variance between cases and controls using Jenson-Shannon divergence dissimilarity in terms of bacteria (B) and fungi (D). R^2 and *p* value statistics in the β-diversity panels are based on PERMANOVA analysis.

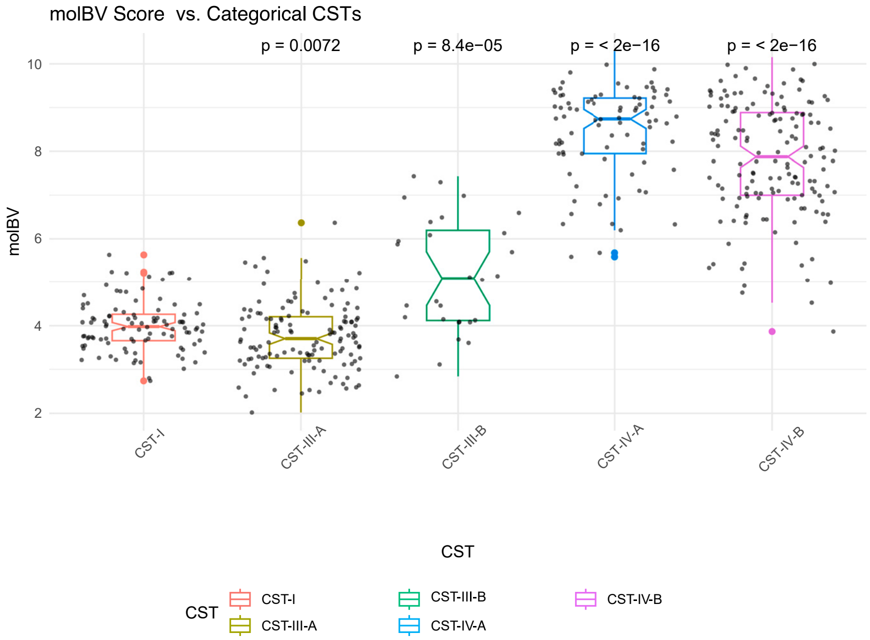

Figure S2. Distribution of *molBV* Scores in cervicovaginal CST categories, related to Table 2

Boxplot and violin plot show the distribution of continuous *molBV* Nugent-like scores (y axis) by discrete community state type (CST) (x axis). Values 7–10 are considered to represent an mBV-positive state. *p* values were computed using Wilcoxon rank sum test between CST-I vs. the other CSTs.

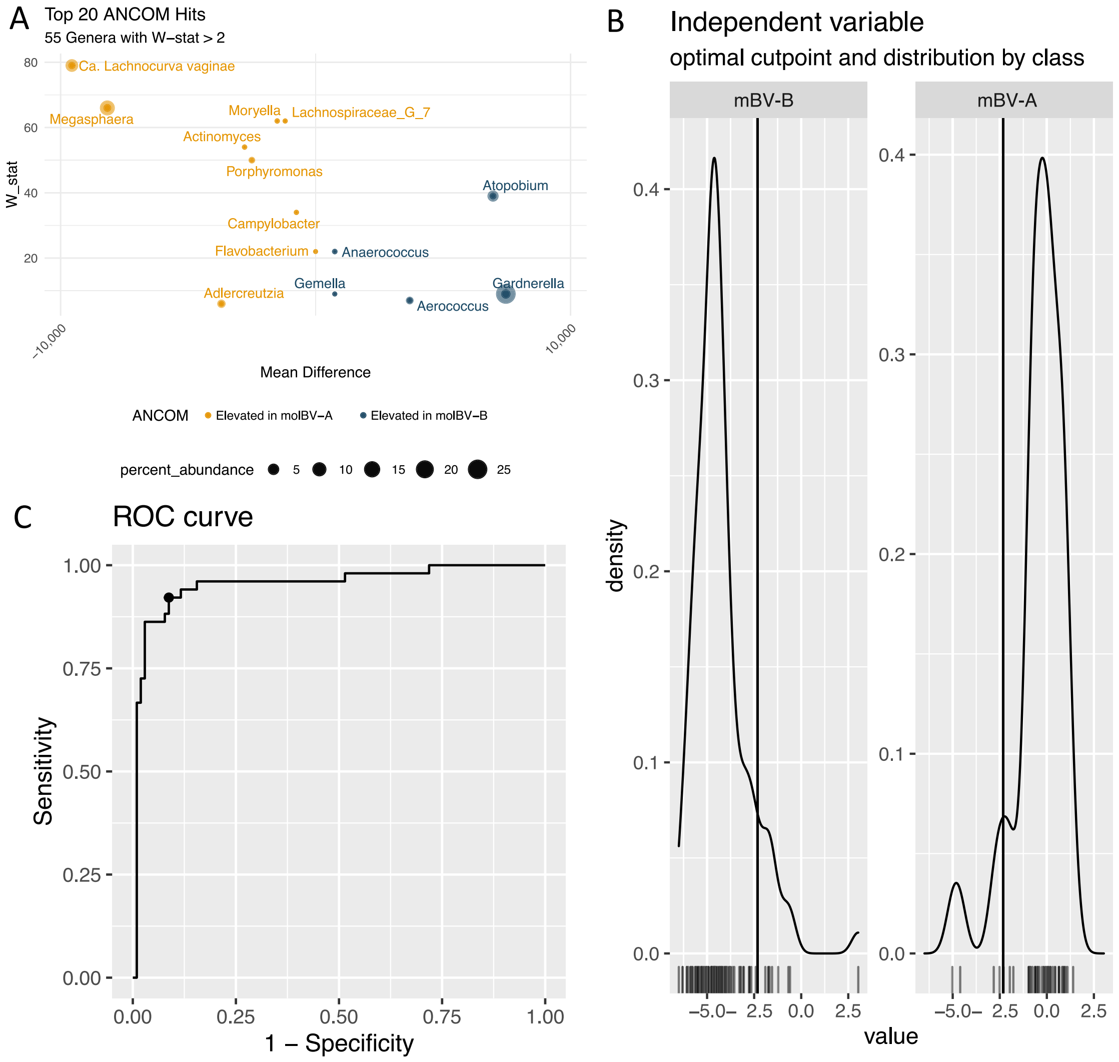

Figure S3. mBV-A vs. mBV-B CVM comparison and prediction using *Ca. Lachnocurva vaginae*, related to Figure 3

(A) Top 20 genera (in terms of W-stat) identified to be differentially abundant between mBV-A and mBV-B groups.

(B) Distribution of *Ca. Lachnocurva vaginae* as a ratio of *Lactobacillus* between mBV-B and mBV-A with the solid line indicating the optimal cut-point for differentiation between the two groups determined using the sum of sensitivity and specificity.

(C) Receiver operating characteristic (ROC) analysis for identifying mBV-A vs. mBV-B using *Ca. Lachnocurva vaginae* (ROC area under the curve [AUC] = 0.93).

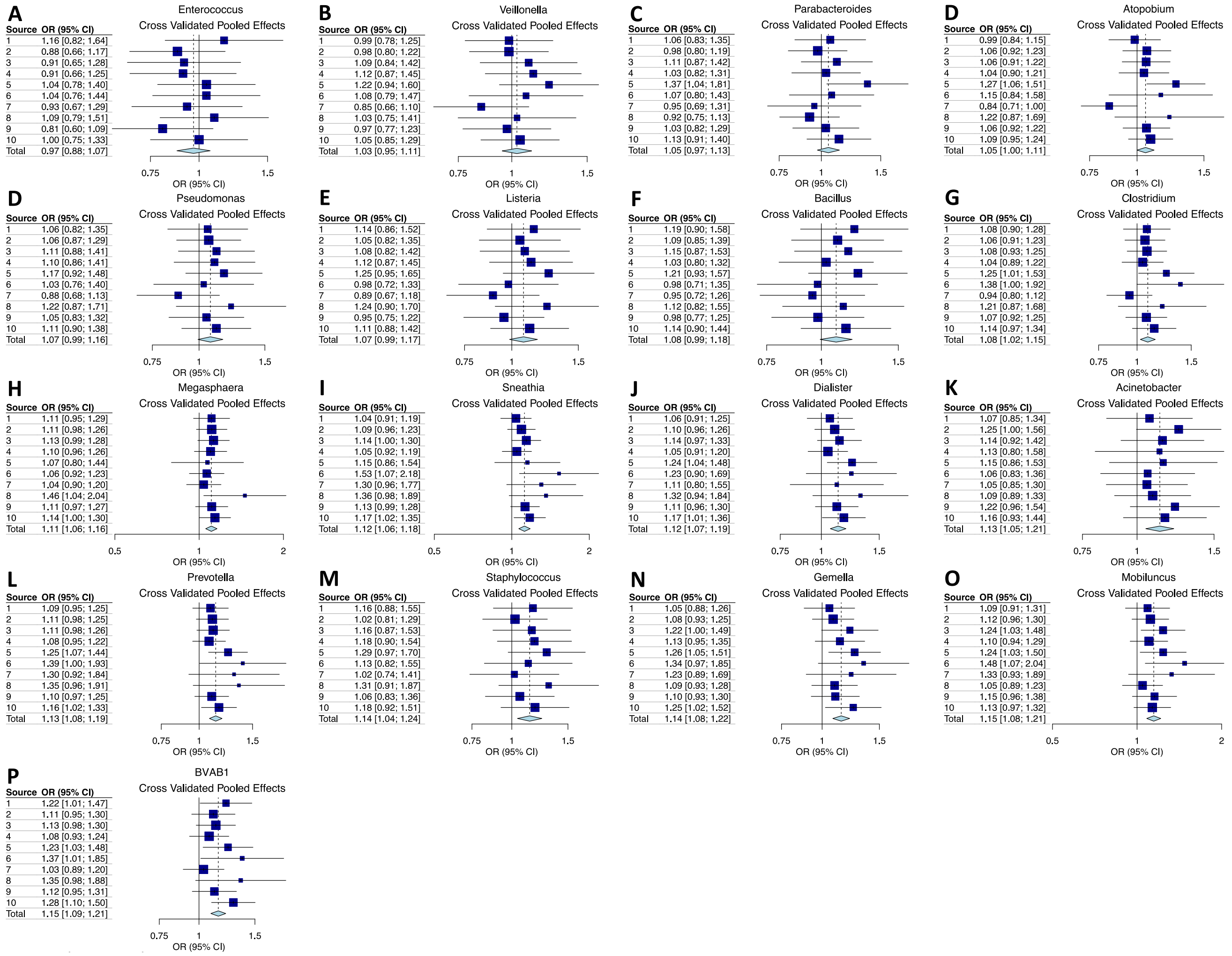

Figure S4. 10-fold Monte Carlo cross-validation of bacterial taxa for CT acquisition at the prospective visit (t_{-1}), related to Figure 3A

(A–P) Results of the 10-fold Monte Carlo cross-validation for the bacteria that were found to be significantly associated with prospective CT acquisition using ANCOM. Dark blue squares represent the replication effects in the testing set (a bacterium is included for analysis if at least 1-fold identifies it to be significant after adjustment for FDR < 0.05).

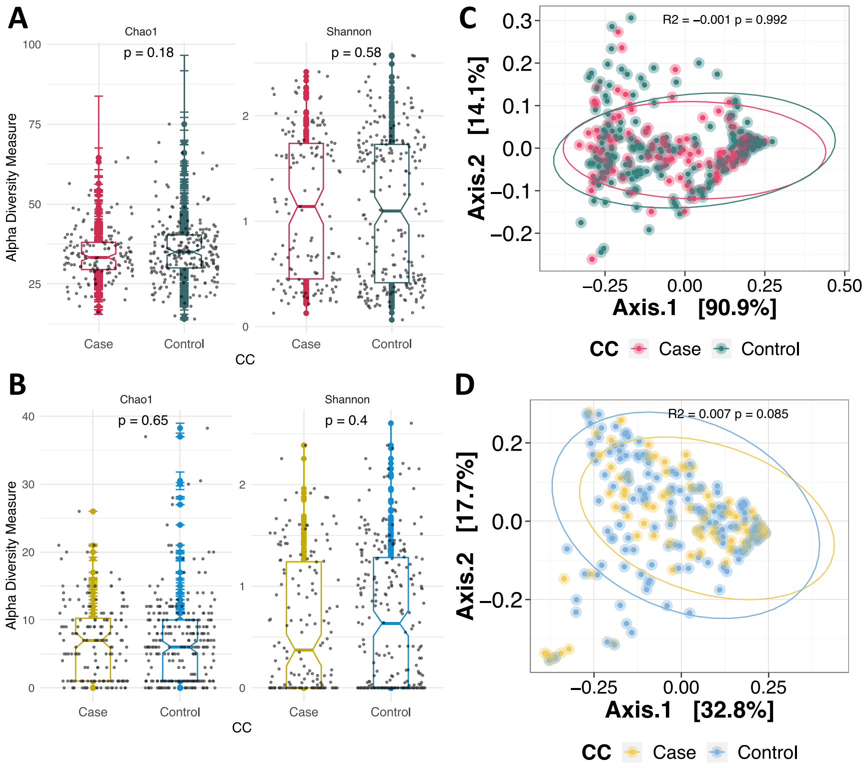

Figure S5. CVM diversity After CT treatment (visit t_{+1}), related to Figure 2 and Table 3

The figure shows the differences in CVM between CT cases after azithromycin or doxycycline antibiotic treatment following incident CT infection and in controls at visit t_{+1}.

(A and B) The α diversity for the bacterial and fungal communities, respectively, with *p* values within each plot calculated using Wilcoxon rank sum test.

(C and D) PCoA for bacteria and fungi, respectively. R^2 and *p* values were calculated using PERMANOVA.

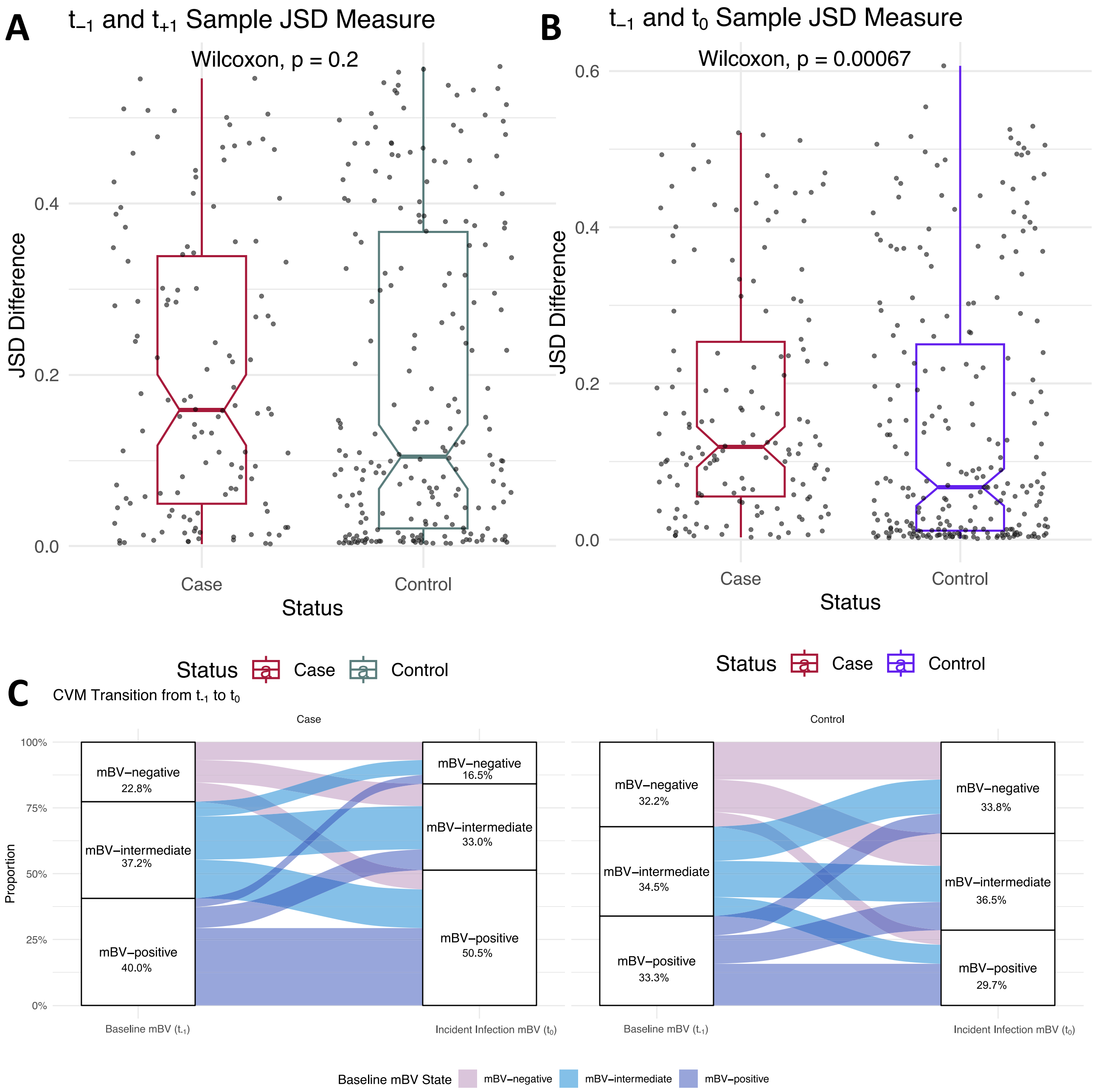

Figure S6. Transition metrics of the CVM between visits, related to Table 3, model B

(A and B) The Jensen-Shannon divergence (JSD) difference between the pre-infection (t_{-1}) to post-infection (t_{+1}) CVM and the pre-infection (t_{-1}) and incident infection (t_0) CVM, respectively. Cases and controls are calculated separately and displayed in colors as indicated below the figure. JSD distances are on the y axis and represent the β diversity with 0 indicating perfect similarity and 1 indicating complete difference in composition of the CVM.

(C) Alluvial plots between t_{-1} and t_0 visits that illustrate the transition between mBV states in cases (left plot) as a result of incident CT infection (t_0) and in controls (right plot) over a similar time period.

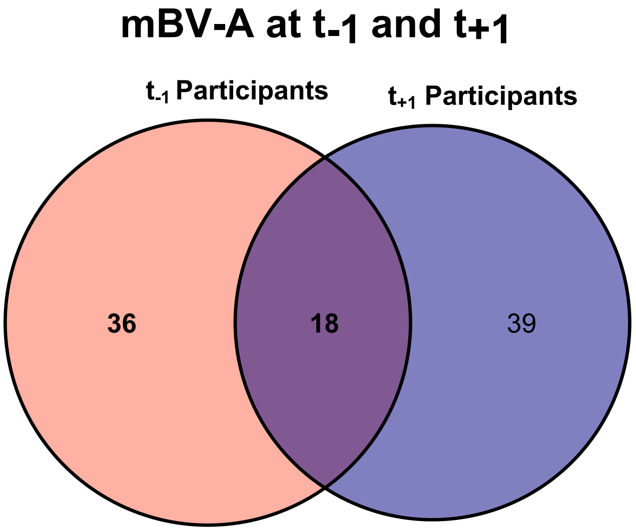

Figure S7. Venn diagram showing mBV-A subtype overlap between t_{-1} and t_{+1} visits in participants with post- t_{+1} CT follow-up data, related to Table 3, model B

Venn diagram shows number of participants and the overlap of participants with the mBV-A subtype at the t_{-1} and t_{+1} visits, as well as the number of participants that had this mBV subtype in both visits.

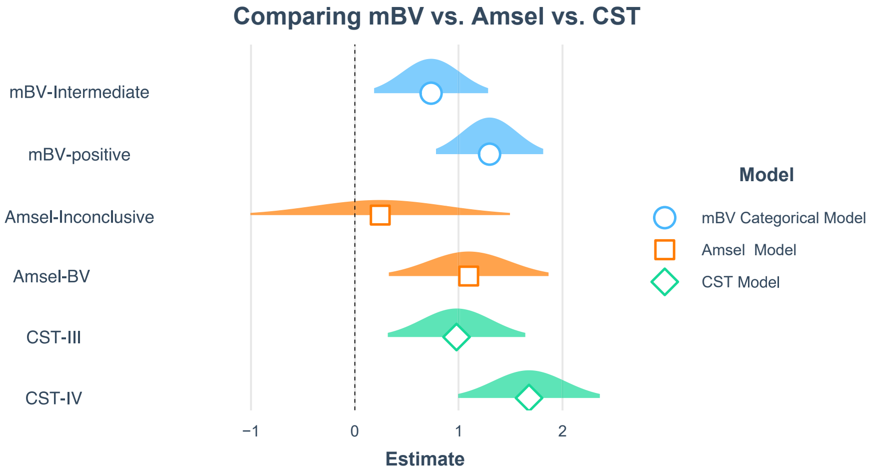

Figure S8. Sensitivity analysis assessing performance of CVM measures, related to STAR Methods

Figure shows the effect estimates of the CVM measures available within the Mt. Sinai cohort (i.e., mBV, Amsel, and CSTs) with incident CT at t_0 being the outcome with adjustment for SRBS, HR-HPV status, and school attendance. Effect estimates are shown in the plot with specific values for the model coefficients, standard errors, and *p* values shown in Table S3. Table S3 footnote indicates the AIC and BIC values that were used for model selection.
